# The Construction of an Environmentally Friendly Super-Secreting Strain of *Bacillus subtilis* through Systematic Modulation of Its Secretory Pathway Using the CRISPR-Cas9 System

**DOI:** 10.3390/ijms25136957

**Published:** 2024-06-25

**Authors:** Jordi Ferrando, David Miñana-Galbis, Pere Picart

**Affiliations:** Faculty of Pharmacy and Food Science Technology, Department of Biology, Healthcare and the Environment, Microbiology Section, Universitat de Barcelona, Avinguda Diagonal 643, 08028 Barcelona, Spain; jferranu7@alumnes.ub.edu (J.F.); davidminyana@ub.edu (D.M.-G.)

**Keywords:** *Bacillus subtilis*, CRISPR-Cas9, heterologous protein, secretion pathway, bottleneck

## Abstract

Achieving commercially significant yields of recombinant proteins in *Bacillus subtilis* requires the optimization of its protein production pathway, including transcription, translation, folding, and secretion. Therefore, in this study, our aim was to maximize the secretion of a reporter α-amylase by overcoming potential bottlenecks within the secretion process one by one, using a clustered regularly interspaced short palindromic repeat-Cas9 (CRISPR-Cas9) system. The strength of single and tandem promoters was evaluated by measuring the relative α-amylase activity of AmyQ integrated into the *B. subtilis* chromosome. Once a suitable promoter was selected, the expression levels of *amyQ* were upregulated through the iterative integration of up to six gene copies, thus boosting the α-amylase activity 20.9-fold in comparison with the strain harboring a single *amyQ* gene copy. Next, α-amylase secretion was further improved to a 26.4-fold increase through the overexpression of the extracellular chaperone PrsA and the signal peptide peptidase SppA. When the final expression strain was cultivated in a 3 L fermentor for 90 h, the AmyQ production was enhanced 57.9-fold. The proposed strategy allows for the development of robust marker-free plasmid-less super-secreting *B. subtilis* strains with industrial relevance.

## 1. Introduction

**Figure 1:**
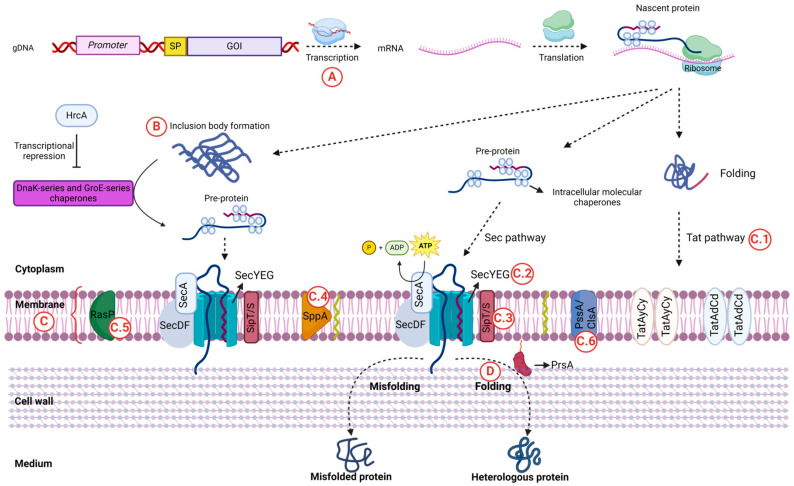
Schematic representation of strategies to overcome the main bottlenecks limiting the efficient expression of a secreted protein in *Bacillus subtilis*. (A) At the transcriptional level, maximum expression of the gene of interest (GOI) is achieved by using strong promoters, optimized signal peptides (SPs), and gene amplification. (B) In the cytoplasm, intracellular GroE and DnaK chaperones prevent protein aggregation. (C) Membrane modification strategies to enhance protein secretion include (C.1) exploiting the Tat pathway; (C.2) overexpressing the SecYEG translocon; (C.3–C.5) optimizing signal peptide processing of signal peptidases SipT and SipS and signal peptide peptidases SppA and RasP; and (C6) cell surface engineering by constructing PssA and ClsA mutants. Lastly, (D) overexpression of extracytoplasmic PrsA aids in proper protein folding and prevents degradation. Created with BioRender.com (accessed on 2 May 2024).

The Gram-positive bacterium *Bacillus subtilis* is widely used as a bacterial workhorse in microbial fermentations for the mass production of heterologous proteins. Several features of this bacteria are advantageous for biotechnological applications, including (i) its generally recognized as safe (GRAS) or qualified presumption of safety (QPS) status; (ii) rapid growth in usually inexpensive media, typically reaching high cell densities in large-scale fermentations; (iii) secretion of recombinant proteins into the culture medium, which simplifies its purification and eliminates the need for cell lysis; and (iv) ease of handling and manipulation, with genetically well-characterized expression systems. Therefore, the high secretion capacity of *Bacillus* species and their close relatives has enabled the development of industrial strains capable of producing enzymes at the scale of several grams per liter [1,2]. However, this capacity for the high-titer production of commercially relevant enzymes is limited by several bottlenecks within the protein production process, spanning from transcription and translation to folding and secretion, thus significantly reducing the overall yield of extracellular enzymes [3,4,5]. Briefly, the main bottlenecks within the secretory pathway of *B. subtilis* can be summarized as follows:

(A) At the transcriptional level, it is a pre-requisite to amplify the expression of the gene of interest, which is commonly achieved using strong or inducible promoters and high-copy-number vectors [6,7]. However, the resulting strains harbor antibiotic resistance markers, which limits their industrial application due to legal restrictions and concerns related to antibiotic usage. Consequently, the construction of environmentally friendly multicopy strains is preferred [8,9,10]. (B) In the cytoplasm, the overexpressed proteins are prone to forming insoluble aggregates and inclusion bodies, rendering the protein inactive. By promoting the proper folding of proteins, which thus retain their biological activity, the overexpression of the GroE and DnaK series of intracellular chaperones has resulted in an enhanced expression of many enzymes [11,12,13]. (C) Within the membrane, the overexpression of exoproteins can cause jamming of the translocation machinery, thereby reducing the yield of secreted proteins. To overcome this barrier, several approaches have been adopted: (C.1) exploitation of the Tat pathway to reroute Sec-dependently secreted enzymes [14,15]; (C.2) overexpression of the translocon SecYEG [16,17]; (C.3) overexpression of the signal peptide processing enzymes SipS and SipT [18]; (C.4) overexpression of the signal peptide peptidase SppA, which cleaves remnant signal peptides left in the cell membrane after the action of SipS and SipT [19]; (C.5) overexpression of the signal peptide peptidase RasP, which prevents perturbation of the membrane and cell envelope [20]; and (C.6) cell surface engineering by modulating the expression of relevant cell surface enzymes, such as phosphatidylserine synthase (PssA) and cardiolipine synthase (ClsA), which have been shown to dramatically increase the overall presence of anionic membrane phospholipids, thus eliciting a higher secretion of enzymes [21]. (D) Once the membrane has been crossed and the extracytoplasmic compartment reached, overexpression of the major extracytoplasmic folding factor PrsA prevents proteolytic degradation while facilitating proper folding, thus enhancing the production of recombinant proteins [18,22]. A schematic representation of the aforementioned steps for the efficient expression of secreted proteins is depicted in Figure 1.

Intensive efforts have been made to surmount these limitations, focusing primarily on the engineering of expression vectors to allow for the overexpression, deletion, or genetic modification of individual steps in the overall production of specific extracellular recombinant proteins [23,24]. Although significant improvements have been reported, in many cases targeting specific genes, the use of plasmids, expensive inducers, and antibiotic selection markers hampers the application of the newly engineered strains to large-scale fermentation. Here, we outline for the first time a systematic analysis encompassing CRISPR-Cas9 genome editing modifications of most of the genes involved in the secretion of extracellular proteins, from transcription and translation to folding and secretion. As its expression is easy to measure, the α-amylase AmyQ was chosen as a readout to assess the effect of each genetic modification, with the ultimate goal of constructing a stable, environmentally friendly *B. subtilis* producer strain with practical application to industry.

Firstly, saturating levels of the specific *amyQ* message were reached by inserting six gene copies at ectopic sites within the *B. subtilis* chromosome under the control of a strong synthetic promoter. Secondly, we consecutively targeted various components of the post-transcriptional machinery, focusing on chaperones, folding processes, translocon systems, membrane stress factors, and metabolic loads, aiming to maximize the ability of *B. subtilis* to secrete the extracellular α-amylase AmyQ. Using this approach, we successfully debottlenecked the exoprotein secretion route at every level, resulting in a 26-fold increase in AmyQ expression. The ability of the newly constructed strain to produce the α-amylase AmyQ was verified using a 3 L fermentor, showing that the super-secreting engineered strain of *B. subtilis* holds commercial potential. We believe this work can provide a better understanding of the cellular mechanisms in the protein production pathway of this commonly used expression host and may offer some promising avenues for heterologous protein secretion in *B. subtilis*.

## 2. Results

### 2.1. The Effect of Single and Tandem Promoters on the Expression of Recombinant AmyQ α-Amylase in B. subtilis

**Figure 2:**
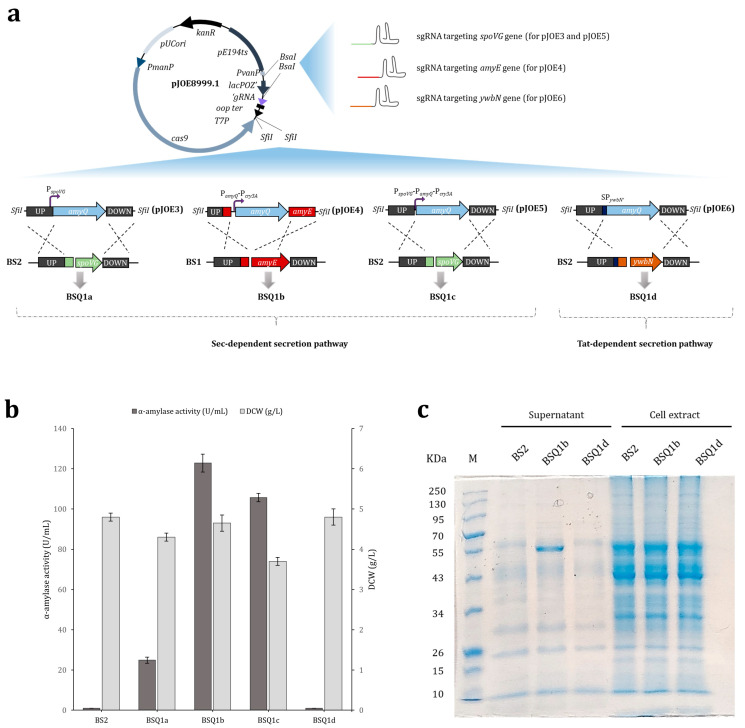
Construction, expression, and secretion of AmyQ α-amylase in strains BSQ1a, BSQ1b, BSQ1c, and BSQ1d. (**a**) Schematic representation of the BSQ1a-BSQ1d strain construction system using plasmids pJOE3-6. The 20 nt guide sequence targeting *spoVG* (for pJOE3 and pJOE5), *amyE* (for pJOE4), and *ywbN* (for pJOE6) was inserted into the *BsaI* sites of pJOE8999.1. The homologous repair template for each plasmid was inserted using the *SfiI* site. The BSQ1a strain harbors an *amyQ* copy at the *spoVG* locus under the control of the *spoVG* promoter (P*_spoVG_-amyQ*). BSQ1b possesses an *amyQ* copy placed at the *amyE* locus under the control of double promoter P*_amyQ_*-P*_cry3A_*. In BsQ1c, the *amyQ* gene is inserted at the *spoVG* locus under the control of triple promoter P*_spoVG_*-P*_amyQ_*-P*_cry3A_*. Integration of the *amyQ* gene containing the YwbN’ signal peptide at the *ywbN* site yielded strain BSQ1d. In this strain, AmyQ secretion occurred through the Tat secretion pathway, whereas strains BSQ1a-c secreted AmyQ through the Sec pathway. (**b**) Extracellular AmyQ activity and dry cell weight (DCW) in engineered *Bacillus subtilis* BSQ1a-BSQ1d strains. BS2 was used as a control strain. The error bars represent the average ± standard deviation of three biological replicates. (**c**) SDS-PAGE analysis of supernatants and cell extracts derived from strains BS2, BSQ1b, and BSQ1d. The expected molecular weight of 55 kDa for AmyQ protein is indicated by the arrow. Lane M shows the molecular weight marker.

The parental *B. subtilis* strain (BS1) inherently possesses a functional *amyE* gene, which encodes an extracellular amylase. Thus, we began by knocking out this gene to render the resulting BS2 strain inactive for amylase production (Figure S1a), thereby avoiding interference with the α-amylase screening method. Subsequently, BS2 was used as the initial strain for genetic manipulation. One of the most cost-effective and efficient methods to achieve high production of recombinant proteins is optimization of the promoter at the transcriptional level, as this element enables gene expression and regulation [10,25,26]. Maximizing the gene expression commonly involves using constitutive promoters. Accordingly, we initially selected the strong *spoVG* promoter to drive the expression of the α-amylase *amyQ* gene, as this widely used promoter is capable of delivering high-level gene expression in *B. subtilis* [27]. Thus, using the pJOE8999.1 plasmid as a backbone [28] and specific primers (Table S1), the pJOE3 vector was engineered for the in-frame replacement of the *spoVG* gene from strain BS2 with the *amyQ* gene, placing its expression under the control of the *spoVG* promoter (P*_spoVG_*-*amyQ*) in strain BSQ1a (Figure 2a). The strength of this promoter was evaluated by culturing BSQ1a in a production medium at 37 °C and 220 rpm for 48 h, which resulted in α-amylase secretion into the medium of about 24.86 ± 1.53 U/mL (Figure 2b). This value served as a reference (control) for further analysis.

Next, we explored the possibility of using two or more tandem promoters to further improve the expression levels of the *amyQ* gene, as this strategy has been shown to be effective [29,30,31]. Thus, the dual tandem promoter P*_amyQ_*-P*_cry3A_*, composed of the engineered *amyQ* promoter and the *cry3A* promoter (Figure S2), and the triple tandem promoter P*_spoVG_*-P*_amyQ_*-P*_cry3A_*were constructed using specific primers (Table S1) and inserted into the backbone vector pJOE8999.1 to yield plasmids pJOE4 and pJOE5, respectively. The two constructs were subsequently inserted into the *B. subtilis* chromosome to obtain the recombinant strains BSQ1b (P*_amyQ_*-P*_cry3A_*-*amyQ*) and BSQ1c (P*_spoVG_*-P*_amyQ_*-P*_cry3A_*-*amyQ*). The identity of each recombinant strain was confirmed by PCR and Sanger sequencing (Figure S1b–d). A comprehensive scheme depicting the construction of each AmyQ-producing strain is provided in Figure 2a. The strength of the tandem promoters to drive *amyQ* expression was evaluated by culturing both strains in production media, and the levels of secreted α-amylase were compared with the control. Additionally, the dry cell weight (DCW) of each strain was calculated (Figure 2b). The maximum value of α-amylase activity was 122.8 ± 4.4 U/mL in strain BSQ1b (the P*_amyQ_*-P*_cry3A_* promoter), which was 4.9-fold higher compared to BSQ1a, whereas the α-amylase activity in the BSQ1c strain (the P*_spoVG_*-P*_amyQ_*-P*_cry3A_*promoter) was about 1.2-fold lower than in BSQ1b. Notably, while the DCW of strains BSQ1a and BSQ1b was similar (about 4.3 and 4.7 g/L, respectively), that of BSQ1c was markedly lower (3.7 g/L). Conclusively, the dual promoter P*_amyQ_*-P*_cry3A_* led to the most pronounced expression level of *amyQ* among the promoters investigated here, and consequently, strain BSQ1b was selected for further studies.

### 2.2. Secretory Expression of AmyQ via the Non-Classical (Tat) Secretion Pathway

Previous studies have shown that overexpression of secreted proteins in *B. subtilis* can cause jamming at the cell membrane due to a shortage of Sec pathway components [11,32]. However, *B. subtilis* also possesses the twin-arginine translocation (Tat) system, which facilitates the transport of fully folded proteins across membranes [33,34,35]. Therefore, seeking to harness the Tat pathway as a complementary secretion route, the widely used twin-arginine signal peptide of *B. subtilis* YwbN [14,36,37] was engineered (YwbN’), as described by Yang and coworkers [38], and placed in frame with the *amyQ* gene (strain BSQ1d) (Figure S1e), with the aim of redirecting AmyQ amylase secretion to the Tat pathway. A scheme of the proposed alternative secretion route for AmyQ and the construction of strain BSQ1d is depicted in Figure 2a. Surprisingly, the α-amylase activity assays showed no activity in the culture supernatant of the recombinant BSQ1d strain (Figure 2b). SDS-PAGE analysis revealed a prominent band with the expected molecular mass (55-kDa) for AmyQ in the supernatants of strain BSQ1b, which secretes AmyQ through the Sec pathway, but this band was found in neither the supernatant nor the cell extract of strain BSQ1d (Figure 2c). These results suggest that SP*_ywbN’_* cannot direct the extracellular secretion of AmyQ using the Tat pathway, and the use of this route was therefore discarded for further studies.

### 2.3. Maximizing amyQ Expression by Inserting Multiple amyQ Gene Copies into the B. subtilis Chromosome

**Figure 3:**
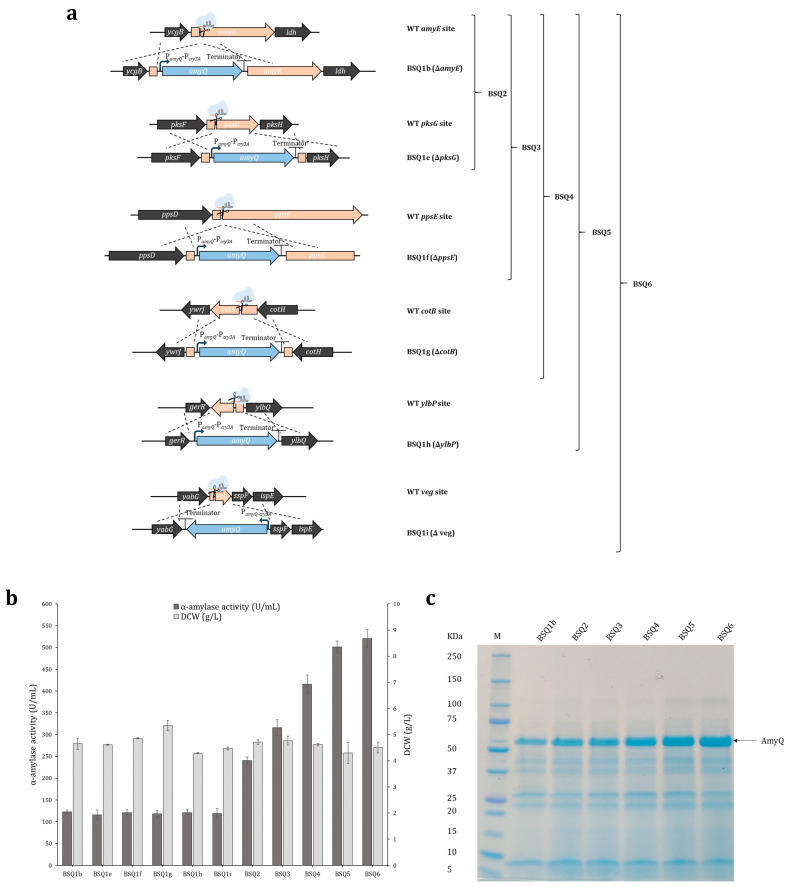
Construction and analysis of *Bacillus subtilis* strains with multiple *amyQ* expression cassette (*amyQ*_Ec) integrations. (**a**) Schematic representation of the construction system for strains BSQ1b, BSQ1e-i, and BSQ2-6. Individual integration of *amyQ*_Ec at the *amyE*, *pksG*, *ppsE*, *cotB*, *ylbP*, and *veg* loci yielded BSQ1b, BSQ1e, BSQ1f, BSQ1g, BSQ1h, and BSQ1i, respectively. Iterative integration of *amyQ*_Ec yielded strains BSQ2 (∆*amyE*, ∆*pksG*), BSQ3 (∆*amyE*, ∆*pksG*, ∆*ppsE*), BSQ4 (∆*amyE*, ∆*pksG*, ∆*ppsE*, ∆*cotB*), BSQ5 (∆*amyE*, ∆*pksG*, ∆*ppsE*, ∆*cotB*, ∆*ylbP*), and BSQ6 (∆*amyE*, ∆*pksG*, ∆*ppsE*, ∆*cotB*, ∆*ylbP*, ∆*veg*) containing two to six *amyQ* copies, respectively. (**b**) Extracellular AmyQ activity and dry cell weight (DCW) in engineered *B. subtilis* strains. The error bars represent the average ± standard deviation of three biological replicates. (**c**) SDS-PAGE showing the protein supernatants from BSQ1b (1), BSQ2 (2), BSQ3 (3), BSQ4 (4), BSQ5 (5), and BSQ6 (6). Supernatants were prepared from cells incubated at 37 °C and 220 rpm for 48 h, cleared by centrifugation, and 15 μL of cleared supernatant proteins was loaded onto the SDS-PAGE gel in each lane. The estimated molecular weight for AmyQ protein of 55 kDa is indicated by an arrow. Lane M corresponds to the molecular weight marker.

To ascertain whether *amyQ* expression could be achieved independently of the integration site, multiple copies of the *amyQ* gene were placed under the control of the previously selected double promoter P*_amyQ_*-P*_cry3A_* and were independently inserted into the BS2 chromosome, one by one. Each expression cassette (P*_amyQ_*-P*_cry3A_*-*amyQ*), hereafter named *amyQ_*Ec, was inserted at ectopic sites within the BS2 chromosome, which were chosen based on their potential to be deleted without affecting strain growth [39,40]. The sets of vectors, integration sites, and primers are provided in the Materials and Methods section, and the resulting strains were named BSQ1e (*amyQ_*Ec at *pksG*), BSQ1f (*amyQ_*Ec at *ppsE*), BSQ1g (*amyQ_*Ec at *cotB*), BSQ1h (*amyQ_*Ec at *ylbP*), and BSQ1i (*amyQ_*Ec at *veg*) (Figure 3a). Enzymatic assays and DCW analysis showed that each of the strains harboring one copy of *amyQ_*Ec secreted similar levels of α-amylase to the medium, with activity values of 115.5–122.8 U/mL. This indicates that the integration of *amyQ_*Ec at the selected ectopic sites did not affect its expression. Similarly, comparable DCW values were obtained for all strains (Figure 3b). Overall, we concluded that *amyQ_*Ec was uniformly expressed regardless of the integration site.

The first dedicated step in the protein secretion route is the transcription of a particular gene into mRNA. To achieve maximum expression, one of the most adopted strategies is to amplify the copy number of the gene of interest [8,26,41]. The higher the copy number, the greater the expression, until the point is reached where increasing the gene copy number no longer results in increased expression [9]. In this context, iterative genome editing was performed using successive double, triple, quadruple, quintuple, and sextuple *amyQ_*Ec integration to yield strains BSQ2 (*amyQ_*Ec at *amyE* and *pksG*), BSQ3 (a*myQ_*Ec at *amyE*, *pksG*, and *ppsE*), BSQ4 (*amyQ_*Ec at *amyE*, *pksG*, *ppsE,* and *cotB*), BSQ5 (*amyQ_*Ec at *amyE*, *pksG*, *ppsE*, *cotB*, and *ylbP*), and BSQ6 (*amyQ_*Ec at *amyE*, *pksG*, *ppsE*, *cotB*, *ylbP,* and *veg*). The identity of each recombinant strain was successfully confirmed by PCR and Sanger sequencing (Figure S1f–j). A comprehensive diagram of the multiple integration sites of *amyQ_*Ec in each strain is provided in Figure 3a.

α-amylase assays revealed a continuous increase in the levels of enzymatic activity until the copy number of *amyQ*_Ec reached the value of six. The α-amylase secretion by strain BSQ2 was almost two-fold higher compared to BSQ1b (122.8 ± 4.4 U/mL), reaching a value of 240.4 ± 8.0 U/mL (two-fold; *p* < 0.05). Strains BSQ3, BSQ4, and BSQ5 secreted higher levels of α-amylase compared to strain BSQ2, albeit the rates of increase were lower, reaching values of 315.7 ± 18.4 U/mL (2.6-fold; *p* < 0.05), 415.7 ± 21.1 U/mL (3.4-fold; *p* < 0.05), and 501.2 ± 13.5 U/mL (4.1-fold; *p* < 0.05), respectively. Finally, strain BSQ6 exhibited about 520.6 ± 20.2 U/mL of α-amylase activity (4.2-fold; *p* < 0.05), which was similar to strain BSQ5 (no significant differences; *p* > 0.05), suggesting the levels of the *amyQ*-specific message had saturated the post-transcriptional machinery of this strain (Figure 3b). SDS–PAGE analysis of the supernatants from the various strains (Figure 3c) showed an increasingly prominent band of 55 kDa, which corresponds to the molecular weight of AmyQ. Overall, the levels of α-amylase activity were notably increased, being 20.9-fold higher in BSQ6 than the values observed in the initial BSQ1a strain (Table 1).

### 2.4. Enhancing AmyQ Secretion in the BSQ6 Strain through Genome Modification of the Sec Pathway and Other Potential Post-Transcriptional Bottlenecks

**Figure 4:**
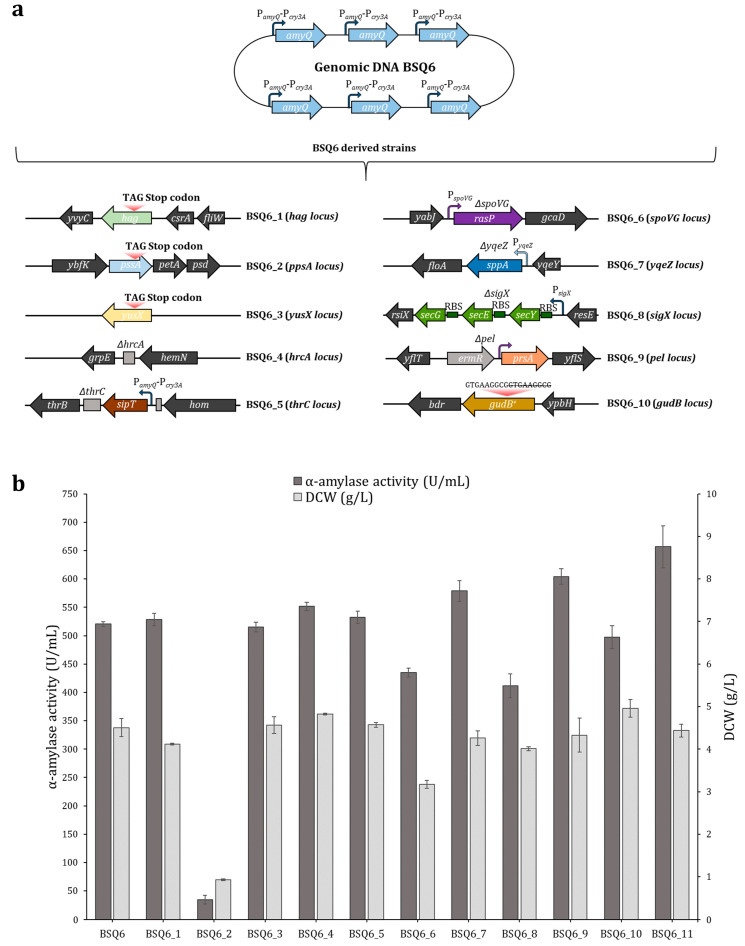
Expression and secretion of AmyQ α-amylase by *Bacillus subtilis* BSQ6 variants. (**a**) Depiction of the multiple BSQ6 mutant strains. Strains BSQ6_1 to BSQ1_3 possess a truncated copy of the *hag*, *pssA*, and *yusX* genes, respectively, achieved by adding a premature stop codon. Strain BSQ6_4 has a clean deletion of the *hrcA* gene. Strains BSQ6_5 to BSQ6_9 were constructed to contain an extra copy of the *sipT*, *rasP*, *sppA*, *secYEG*, or *prsA* genes, respectively, under the control of strong promoters. A functional *gudB* gene was restored in strain BSQ6_10 by deleting 9 bp. Strain BSQ6_11 with combinational overexpression of *sppA* and *prsA*. RBS (ribosome binding site). (**b**) Extracellular AmyQ activity and dry cell weight (DCW) in engineered *B. subtilis* strains. The error bars represent the average ± standard deviation of three biological replicates. BSQ6 was used as a control.

Previous studies have indicated that saturating levels of mRNA expression may potentially lead to the saturation of the secretory machinery, thus hindering effective protein secretion [26]. In this scenario, it has been shown that the overexpression of some components of the secretory machinery can facilitate the secretion and proper folding of the protein of interest [3,11,42]. Therefore, to comprehensively circumvent the potential bottlenecks lying downstream of transcription, we adopted an approach based on targeting several post-transcriptional constraints and evaluated the effect of these mutations in strain BSQ6. Hence, a set of strains harboring genome modifications within the Sec pathway secretion route were constructed using the CRISPR-Cas9 system. Firstly, a truncation or deletion of the *hag*, *pssA*, *yusX*, or *hrcA* genes was independently performed in BSQ6 to obtain strains BSQ6_1, BSQ6_2, BSQ6_3, and BSQ6_4, respectively. Secondly, the insertion of an extra copy of the signal peptidase *sipT* gene at the *thrC* site, placed under the control of the dual promoter P*_amyQ_*-P*_cry3A_*, led to the construction of strain BSQ6_5. Thirdly, the in-frame replacement of the *spoVG*, *yqeZ*, *sigX*, and *pel* genes with *rasP*, *sppA*, the operon *secYEG*, and *prsA* allowed for the overexpression of these genes in strains BSQ6_6, BSQ6_7, BSQ6_8, and BSQ6_9, respectively. Finally, strain BSQ6_10 was designed to contain a functionally active GudB protein. To this end, a 9 bp direct repeat, which produced a cryptic product in strains derived from *B. subtilis* 168, was deleted within the wild-type *gudB* gene sequence [43]. Figure 4a provides a comprehensive schematic representation of the multiple modifications in strain BSQ6. The identity of each recombinant strain was verified by PCR and Sanger sequencing (Figures S1k–r and S3).

The ability of the BSQ6 strain mutants to secrete α-amylase into the media and their DCWs were individually evaluated using shake-flask cultures (Figure 4b). Only two of the ten mutants analyzed, BSQ6_7 (harboring an extra copy of the *sppA* gene) and BSQ6_9 (harboring an extra copy of *prsA*), exhibited significantly enhanced *amyQ* expression, with 1.1- and 1.2-fold higher AmyQ secretion compared to strain BSQ6 (both *p* < 0.05), respectively (Table 1). Instead, BSQ6_6 (harboring an extra copy of the *rasP* gene) and BSQ6_8 (harboring an extra copy of the operon *secYEG*) showed reduced levels of α-amylase activity, both being 1.2-fold lower than the parental strain. Notably, strain BSQ6_2 (with a truncated copy of the *pssA* gene) showed a prominent growth defect, and therefore the levels of α-amylase secreted into the media were dramatically reduced. The rest of the mutants displayed values of α-amylase activity similar to the control BSQ6 strain (all *p* > 0.05) (Figure 4b).

Subsequently, to further develop our *B. subtilis* strain as a high expressor of the AmyQ α-amylase, we investigated whether the double mutant BSQ6_11, carrying both beneficial mutations (overexpression of *sppA* and *prsA*), could enhance AmyQ production. As detailed in Table 1, BSQ6_11 displayed outstanding secretion of the AmyQ α-amylase, with a remarkable 26.4-fold increase compared to the initial strain BSQ1a.

### 2.5. Scale-Up of α-Amylase Production in a 3 L Fermentor

**Figure 5:**
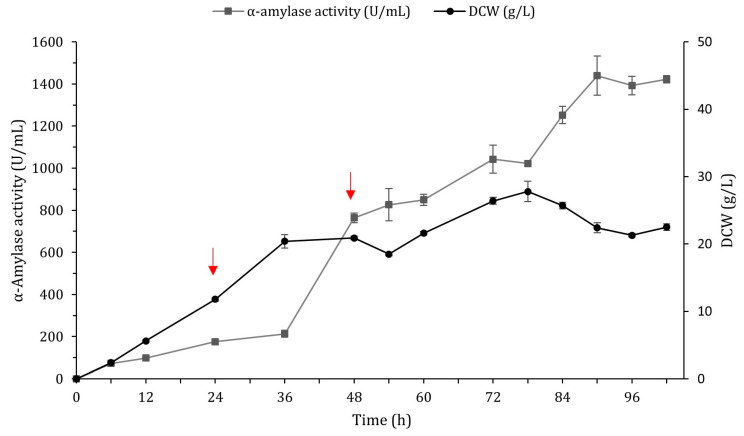
Production of α-amylase AmyQ in recombinant strain BSQ6_11 in 3 L fermentor. AmyQ production and DCW were monitored as a function of time. Solid square: α-amylase activity in medium. Solid circle: DCW. Red arrows indicate pulse feedings at 24 and 48 h. Error bars indicate the standard deviation from the mean of the three experimental data replicates.

The expression efficiency of BSQ6_11 was further explored in a 3 L fermentor. The fermentor was inoculated with 4% (*v*/*v*) of freshly cultured BSQ6_11 grown in production medium at 37 °C for 18 h. To maintain cell growth and α-amylase production, we chose a fed-batch strategy with pulse feeding of highly concentrated sucrose (250 g/L and 580 g/L) and soy peptone (250 g/L), which were fed intermittently in two pulses of 0.12 L in response to an increased DO signal. During the growth phase (Figure 5), the maximum biomass in the fermentor reached a DCW of 27.8 g/L at 78 h. The activity of α-amylase in the medium was continuously increased and reached a maximum of 1439.2 ± 92.7 U/mL at 90 h, with a productivity of 16 U/mL h. This was 2.2-fold greater than the α-amylase activity in the fermentation supernatant of the same BSQ6_11 strain in the shake-flask cultures and corresponded to a remarkable 57.9-fold higher AmyQ secretion compared to the parental strain BSQ1a (Table 1). The high activity of α-amylase indicated that the engineered strain BSQ6_11 was a suitable host for the industrial production of the AmyQ α-amylase.

## 3. Discussion

Despite the extensive knowledge available on the secretion of enzymes by *B. subtilis*, we believe there is scope for improving its capacity to overproduce commercially significant enzymes. The *B. subtilis* secretory pathway can be divided into three stages: transcription, translocation and folding, and secretion [44]. Along this pathway, each step is a potential bottleneck for high-level production, and these restrictions should be identified if the yields of heterologous proteins are to be improved. Here, we used the CRISPR-Cas9 system to systematically tackle, one by one, the different steps throughout the secretion route of *B. subtilis*. By employing this approach, it was possible to pinpoint targets whose modification could enhance the capacity of the secretion machinery, thus maximizing the secretion of the reporter AmyQ α-amylase in *B. subtilis*, as well as constructing an environmentally friendly super-secreting *B. subtilis* strain with practical application to industry.

To enhance or appropriately adjust the gene expression levels in *B. subtilis*, it is essential to study the promoters that regulate transcription levels, as they constitute one of the most important elements for facilitating the high production of recombinant proteins [45,46]. Thus, we evaluated the capacity of single, double, and triple promoters to dictate the expression of the AmyQ α-amylase and found the expression strength of the double promoter (P*_amyQ_*-P*_cry3A_*) to be the greatest (Figure 2b). This outcome was probably due to the presence of mRNA stabilization sequences, which have been shown to increase the expression of industrial enzymes [47,48]. As strength is not necessarily correlated with the number of copies of the promoter [45], the weakness of P*_spoVG_*-P*_amyQ_*-P*_cry3A_* might have been caused by aligning more than two promoters [49]. Additionally, differences in origin can influence the cooperativity of the tandem promoter.

Once a suitable promoter has been chosen, achieving the maximum secretion of a particular enzyme in *B. subtilis* almost always requires the amplification of the gene copy number. This is accomplished by either using high-copy-number replicative plasmids or inserting multiple copies of the desired gene into the chromosome [8,9]. Hence, environmentally friendly, plasmid-less, marker-free multicopy-*amyQ* strains were constructed by sequentially inserting up to six *amyQ* gene copies under the control of the P*_amyQ_*-P*_cry3A_* promoter at ectopic sites within the *B. subtilis* chromosome. As shown in Table 1, the higher the copy number of the *amyQ*_Ec cassette, the higher the *amyQ* gene expression, until at the sixth copy, the levels of α-amylase activity were similar to those achieved with five copies. Comparably to our results, the group of Altenbuchner showed that the integration of five copies of the β-glucosidase *ganA* into the chromosome of *B. subtilis* was required to achieve the maximum expression [9], whereas in the case of the protease *aprL*, only one copy was sufficient [26]. This discrepancy might stem from a lower translation initiation rate owing to different gene sequences downstream of the start codon [50], indicating that the number of copies necessary to achieve the maximum expression is variable and depends on each specific gene. In any case, at this point, we could conclude that the levels of *amyQ*-specific mRNA had saturated the post-transcriptional machinery, and the bottleneck for expression had shifted downstream of transcription.

To elucidate which other factors might impede the AmyQ production process, we targeted essential genes involved in the post-transcriptional stage and non-essential Sec pathway components, aiming to improve the secretion of recombinant AmyQ in *B. subtilis*. After transcription, the first step toward the successful secretion of the newly formed pre-protein in *B. subtilis* requires the action of intracellular chaperones that prevent inappropriate folding or aggregation of the pre-protein within the cell [51]. Previous reports show that inactivation of the repressor HrcA enables the overexpression of intracellular chaperones [52], and the secretion of the AmyS α-amylase has been significantly enhanced using this approach [13]. However, contrary to expectations, *hrcA* deletion exerted no significant effect on AmyQ production and therefore cannot be considered a bottleneck in our *B. subtilis* α-amylase production system.

*B. subtilis* possesses a well-developed and highly efficient Sec pathway that is commonly exploited for the production of industrial enzymes. However, an alternative system, the Tat pathway, exists to facilitate the transport of proteins that fold too tightly or rapidly in the cytosol and compatibility with the Sec pathway [53]. The nature of the signal peptide directs the nascent protein to the Sec or Tat translocase, which is efficiently cleaved by signal peptidases [54] prior to the export of the protein to the extracytoplasmic compartment. Here, we used the signal peptide of the typically Tat-secreted YwbN protein [34,55] to evaluate whether this route was compatible with AmyQ α-amylase secretion, which we hoped would be enhanced via both routes simultaneously. Although successful secretion of alkaline α-amylase has been achieved using an engineered Tat-dependent YwbN signal peptide [38], its application in our study resulted in no α-amylase activity being detected in the extracellular media (Figure 2b). Our findings are in agreement with previous reports pointing to Tat incompatibility with AmyL α-amylase secretion, probably due to the inability of the protein to fold in the cytoplasm, a prerequisite for Tat secretion [14].

High gene expression levels may result in the saturation of the Sec translocon capacity, which may be due to a shortage of translocons or the unavailability of signal peptidases [18,21]. Therefore, the effect of both artificial *secYEG* operon overexpression and signal peptidase *sipT* overexpression on AmyQ secretion was evaluated. While the extra copy of the *sipT* gene resulted in a slight 1.02-fold improvement in AmyQ production, the overexpression of *secYEG* unexpectedly diminished α-amylase activity (Figure 4). Although increasing the number of translocons [16] and the overexpression of SipT [18] can be accompanied by a concomitant increase in the amount of exoenzymes, in other cases, these modifications have had a negligible or even a reducing effect [11], which indicates they have an inconsistent impact on amylase secretion. In the present study, we verified that the translocon SecYEG and the signal peptidase SipT have different effects on the secretion of different proteins.

Bottlenecks in the late stages of protein secretion in *B. subtilis* include the extracellular chaperone prsA [18] and the signal peptide peptidases SppA and RasP [20,56]. Gratifyingly, PrsA overexpression significantly enhanced AmyQ production, with the values of secreted α-amylase exceeding those of BSQ6 by 16%. This result is in line with those of previous studies that have highlighted PrsA as a primary bottleneck in α-amylase secretion, as it facilitates the folding process of mature proteins into a stable and active conformation [11,18,22]. Additionally, the insertion of an extra copy of the *sppA* gene under the control of the strong promoter *yqeZ* [57] significantly increased the α-amylase activity by 11% compared to BSQ6 (Figure 4). This is in agreement with a previous study, which found that the overexpression of *sppA* enhanced the production of α-amylase in *Bacillus licheniformis*, suggesting that this peptidase is necessary for the efficient processing of cleaved signal peptides and to maintain the proper secretion of mature proteins across the membrane under conditions of hyper-secretion [19]. Intriguingly, the overexpression of RasP severely impaired the secretion of AmyQ, in contrast with previous research in which its overexpression markedly improved the secretion of the AmyAc α-amylase [20]. Our hypothesis is that excessive RasP may have some unknown harmful effects on the physiological characteristics of *B. subtilis*, resulting in a significant drop in α-amylase production.

Acquiring a systematic and thorough understanding of Sec pathway components and non-associated Sec pathway proteins that might facilitate target protein secretion could be the key to significantly increasing the yield of recombinant proteins. Indeed, besides optimizing the Sec-related components, this study aimed to further engineer the *B. subtilis* host as a chassis to improve the production of the AmyQ α-amylase. To achieve this, firstly, the *B. subtilis* cell surface was modified by knocking out the *pssA* gene. Previous reports have suggested that the deletion of this gene results in the increased presence of anionic membrane phospholipids in *B. subtilis*, leading to a higher amount of α-amylase being released into the medium [21]. Conversely, *pssA* deletion caused severe growth arrest, indicating that this gene is vital for maintaining the viability of *Bacillus* cells under the conditions of high *amyQ* gene expression. Secondly, reduced expression of the oligoendopeptidase YusX or YusZ has been associated with high secretion levels of enzymes in *B. subtilis* [58]. Nonetheless, the ability to secrete AmyQ in Δ*yusX* mutants remained stable, indicating a negligible effect.

Lastly, we wanted to ascertain whether knocking out specific genes to increase the growth rate of *B. subtilis* would result in higher productivities, as previously reported [39]. To investigate this hypothesis, two strategies were adopted. On the one hand, we examined the effect of deleting the non-essential *hag* gene on the growth rate of BSQ6. The rationale for this approach was that the metabolic energy wasted on expressing the subunit flagellin protein (*hag* gene), one of the most highly expressed genes in the *B. subtilis* genome [59], could be redirected toward the synthesis of α-amylase protein and essential genes, resulting in an increased growth rate, as described in previous studies [60]. However, no significant differences in the growth rate or α-amylase production were observed in BSQ6_1 lacking the *hag* gene. This is not surprising considering that metabolism self-regulation by the cell can often minimize the performance of knock-out strains [61]. On the other hand, we wanted to re-establish the activity of the *gudB* gene, encoding glutamate dehydrogenase (GDH), a gene truncated in the strain *B. subtilis* 168 and its derivatives. As our production medium contained soy peptone, which is rich in amino acids of the glutamate family, we speculated that restoring GudB activity would allow for more efficient utilization of nitrogen sources and therefore confer a growth advantage compared to cells lacking a functional enzyme [43]. However, no significant differences between the strains were observed. As *B. subtilis* carries an additional GDH enzyme encoded by the *rocG* gene, our hypothesis is that although soy peptone is rich in glutamate, the final concentration used in this study may not have been high enough for an additional GDH enzyme to be advantageous [62].

In this work, two pulses of fresh nutrients (sucrose and peptone) proved to be optimal for enhancing AmyQ production in a 3 L fermentor. In pulse fed-batch fermentation, the substrate concentration is kept within certain limits by the pulses to meet the requirements of *B. subtilis* metabolism, thus avoiding starvation and directing the energy obtained from the substrates towards both maintaining cellular metabolism and producing the α-amylase AmyQ. Accordingly, this strategy resulted in a 2.2-fold enhancement of α-amylase activity (1439 U/mL) in comparison to in the shake flasks (657 U/mL). Remarkably, the level of secreted AmyQ reached at the fermentor stage was higher than the value of 1100 U/mL for AmyQ α-amylase activity achieved in previous studies by using the high-copy pKHT10 plasmid in *B. subtilis* [63,64].

This is the first report that describes an improvement in α-amylase extracellular production levels in *B. subtilis* by merely using the CRISPR-Cas9 system, rendering an industrial strain devoid of plasmids and antibiotic selection markers and bypassing the need for expensive inducers. Although the impact of gene modifications within the secretory pathway of heterologous proteins might be variable, probably depending on each specific gene, we consider the strategy presented in this work to obtain the maximum secretion levels from multiple copy gene insertion, along with combinational Sec pathway analysis, a promising approach that will facilitate the construction of robust, ecologically safe, industrial strains of *B. subtilis* in forthcoming years.

## 4. Materials and Methods

### 4.1. Bacterial Strains, Plasmids, and Culture Conditions

All the bacterial strains and plasmids used in this study are listed in Table 2 and Table 3, respectively. *Escherichia coli* DH5α served as the host for cloning and plasmid preparation. Chemically competent *E. coli* strains were prepared and the transformation protocol performed as described previously [65]. *B. subtilis* BS1, which is an asporogenous strain (Δ*sigF*) with reduced lysis (Δ*lytC*) and deficient in seven extracellular proteases (Δ*nprE*, Δ*aprE*, Δ*epr*, Δ*mpr*, Δ*nprB*, Δ*vpr*, Δ*bpr*), was used as a host for AmyQ expression. *B. subtilis* was transformed according to the method of Yasbin et al. [66], using plasmid DNA isolated from the *rec*^+^ strain *E. coli* Turbo (New England Biolabs, Ipswich, MA, USA). The plasmid pJOE8999.1 [28], an *E. coli*/*B. subtilis* shuttle vector harboring the CRISPR-Cas9 system, was used to edit the *B. subtilis* genome. Transformants of *E. coli* and *B. subtilis* were selected on Luria–Bertani (LB) agar plates at 37 °C, supplemented with appropriate antibiotics. To select plasmids in *E. coli*, kanamycin was used at a final concentration of 30 µg/mL, while for *B. subtilis*, kanamycin was used at a final concentration of 6 µg/mL, as well as erythromycin and lincomycin at 2 µg/mL and 25 µg/mL, respectively. All the strains were incubated under shaking conditions at 200 rpm. All the experiments were repeated at least three times, and mean values were used for comparisons.

### 4.2. DNA Manipulations

Standard molecular techniques were carried out following the standard methods [67]. The genes encoding engineered *prsA* (Figure S4) and the α-amylase *amyQ* gene harboring its native signal peptide and the dual promoter P*_amyQ_*-P*_cry3A_* (Figure S2) were synthesized by NZYtech (Lisboa, Portugal). Chromosomal DNA was extracted from *B. subtilis* strains using the NZY Tissue gDNA Isolation kit). Plasmid DNA was isolated from *E. coli* using the NZYSpeedy Miniprep kit. Digested DNA fragments from agarose gel and amplified DNAs in PCRs were isolated using the NZYGelpure kit. All kits and enzymes were purchased from NZYtech (Lisboa, Portugal). Gibson assembly was performed according to the manufacturer’s instructions (Invitrogen, Waltham, MA, USA). All the DNA constructs were sequenced by Macrogen (Seoul, Republic of Korea).

### 4.3. Construction of the Integration Vectors for amyQ Overexpression and Sec Pathway Modulation

All the integration vectors and primers used in this study are listed in Table 2 and Table S1, respectively. In all cases, the integration vectors were constructed using the pJOE8999.1 plasmid as the parental plasmid and required two consecutive steps: (i) cloning specific sgRNA and (ii) cloning a specific editing template.

### 4.4. Cloning of sgRNA

The design of sgRNA for gene editing of *B. subtilis* was accomplished using the sgRNA Designer tool, provided by the Broad Institute [68]. For sgRNA construction targeting each desired gene, two complementary oligonucleotides were ordered (Macrogen, Seoul, Republic of Korea) with the respective overhangs, annealed, and cloned into the vector pJOE8999.1. In brief, both complementary oligonucleotides were mixed at a final concentration of 10 μM in annealing buffer (10× stock containing 100 mM Tris-HCl pH 7.5, 1 M NaCl, and 1 mM ethylenediaminetetraacetic acid (EDTA) (pH 8)), kept at 98 °C for 5 min, and slowly cooled to room temperature. Then, the annealed oligonucleotides were treated with polynucleotide kinase to phosphorylate the 5’ ends, according to the manufacturer’s instructions (Invitrogen, Waltham, MA, USA), and ligated with the *BsaI*-cleaved and dephosphorylated plasmid pJOE8999.1 to incorporate specific target sequences into the vector.

### 4.5. Cloning of the Editing Templates

In a second step, to construct the editing templates, two homologous arms of a similar length and the desired template to be inserted were separately amplified and then fused together by splicing with overlap extension PCR (SOEing-PCR, Table S2). The resulting PCR product was digested with *SfiI* and ligated into the *SfiI*-digested pJOE8999.1 vector to obtain each editing plasmid.

For the construction of the knock-out plasmids pJOE2, pJOE12, pJOE13, pJOE14, pJOE15, and pJOE20, the corresponding sgRNA and homologous repair template were inserted into the plasmid pJOE8999.1 using the following primers: TS2F/TS2R (sgRNA targeting *amyE*), P2_1F/P2_1R and P2_2F/P2_2R (template for *amyE* deletion); TS12F/TS12R (sgRNA targeting *hag*), P12_1F/P12_1R and P12_2F/P12_2R (template for *hag* deletion); TS13F/TS13R (sgRNA targeting *pssA*), P13_1F/P13_1R and P13_2F/P13_2R (template for *pssA* deletion); TS14F/TS14R (sgRNA targeting *yusX*), P14_1F/P14_1R and P14_2F/P14_2R (template for *yusX* deletion); TS15F/TS15R (sgRNA targeting *hrcA*), P15_1F/P15_1R and P15_2F/P15_2R (template for *hrcA* deletion); and TS20F/TS20R (sgRNA targeting *gudB*), P20_1F/P20_1R and P20_2F/P20_2R (template for *gudB* restoration). In the pJOE12, pJOE13, and pJOE14 editing plasmids, the repair template was designed to remove 6 bp of the native sequence to insert a *XhoI* restriction site and 5 bp of a random sequence, causing gene frameshift mutation and consequently a loss of function. The removal of 9 pb of cryptic *gudB* using pJOE20 restored *gudB* function instead. However, pJOE2 and pJOE15 were designed to produce a partial deletion of the corresponding gene, leaving only a few amino acids intact.

In an analogous manner, the knock-in plasmids pJOE3, pJOE4, pJOE5, pJOE6, pJOE7, pJOE8, pJOE9, pJOE10, pJOE11, pJOE16, pJOE17, pJOE18, and pJOE19 were constructed using the following primers: TS3F/TS3R (sgRNA targeting *spoVG*), P3_1F/P3_1R, P3_2F/P3_2R, and P3_3F/P3_3R (template for P*_spoVG_*-*amyQ* integration), P5_1F/P5_1R, P5_2F/P5_2R, and P5_3F/P5_3R (template for P*_spoVG_*-P*_amyQ_*-P*_cry3A_*-*amyQ* integration), P17_1F/P17_1R, P17_2F/P17_2R, and P17_3F/P17_3R (template for *rasP* integration); TS4F/TS4R (sgRNA targeting *amyE*), P4_1F/P4_1R, P4_2F/P4_2R, and P4_3F/P4_3R (template for P*_amyQ_*-P*_cry3A_*-*amyQ* integration); TS6F/TS6R (sgRNA targeting *ywbN*), P6_1F/P6_1R, P6_2F/P6_2R, and P6_3F/P6_3R (template for SP*_ywbN’_*-*amyQ* integration); TS7F/TS7R (sgRNA targeting *pksG*), P7_1F/P7_1R, P7_2F/P7_2R, and P7_3F/P7_3R (template for P*_amyQ_*-P*_cry3A_*-*amyQ* integration); TS8F/TS8R (sgRNA targeting *ppsE*), P8_1F/P8_1R, P8_2F/P8_2R, and P8_3F/P8_3R (template for P*_amyQ_*-P*_cry3A_*-*amyQ* integration); TS9F/TS9R (sgRNA targeting *cotB*), P9_1F/P9_1R, P9_2F/P9_2R, and P9_3F/P9_3R (template for P*_amyQ_*-P*_cry3A_*-*amyQ* integration); TS10F/TS10R (sgRNA targeting *ylbP*), P10_1F/P10_1R, P10_2F/P10_2R, and P10_3F/P10_3R (template for P*_amyQ_*-P*_cry3A_*-*amyQ* integration); TS11F/TS11R (sgRNA targeting *veg*), P11_1F/P11_1R, P11_2F/P11_2R, and P11_3F/P11_3R (template for P*_amyQ_*-P*_cry3A_*-*amyQ* integration); TS16F/TS16R (sgRNA targeting *thrC*), P16_1F/P16_1R, P16_2F/P16_2R, P16_3F/P16_3R, and P16_4F/P16_4R (template for *sipT* integration); TS18F/TS18R (sgRNA targeting *yqeZ*), P18_1F/P18_1R, P18_2F/P18_2R, and P18_3F/P18_3R (template for *sppA* integration); and TS19F/TS19R (sgRNA targeting *sigX*), P19_1F/P19_1R, P19_2F/P19_2R, P19_3F/P19_3R, P19_4F/P19_4R, and P19_5F/P19_5R (template for *secYEG* overexpression). The latter template was constructed using Gibson assembly following the manufacturer’s instructions.

Due to cloning issues during *prsA* template construction, a different approach based on Gibson assembly was adopted for *prsA* insertion at the *pel* locus. Four different fragments were amplified by PCR, corresponding to the (i) 5’ *pel* homologous region (primers AF/AR); (ii) the erythromycin resistance gene from the pBS2EXylR plasmid (BF/BR primers); (iii) synthetic *prsA* (primers CF/CR); and (iv) the 3’ *pel* homologous region (primers DF/DR). The amplicons were designed to allow Gibson assembly to occur following the manufacturer’s instructions (Thermo Fischer Scientific, Waltham, MA, USA). The resulting reaction mixture was used as a template to amplify the 8 kb fragment using the primers AF/DR, which served as donor DNA for *B. subtilis* transformation [69].

**Table 3:** Plasmids used in this study.

Plasmid	Characteristics	Reference
pBS2EXylRPxylA	Plasmid containing the xylose-inducible promoter/xylose repressor system	[70]
pJOE8999.1	P*_manP_*-*cas9*, pUC, pE194^ts^, kan^r^	[28]
pJOE2	*amyE* gene knock-out plasmid derived from pJOE8999.1	This work
pJOE3	*amyQ* gene (P*_spoVG_*) knock-in plasmid derived from pJOE8999.1. Integration at the *spoVG* locus.	This work
pJOE4	*amyQ* gene (P*_amyQ_*-P*_cry3A_*) knock-in plasmid derived from pJOE8999.1. Integration at the *amyE* locus.	This work
pJOE5	*amyQ* gene (P*_spoVG_*-P*_amyQ_*-P*_cry3A_*) knock-in plasmid derived from pJOE8999.1. Integration at the *spoVG* locus.	This work
pJOE6	*amyQ* gene (SP*_ywbN_*_’_) knock-in plasmid derived from pJOE8999.1. Integration at the *ywbN* locus.	This work
pJOE7	*amyQ* gene (P*_amyQ_*-P*_cry3A_*) knock-in plasmid derived from pJOE8999.1. Integration at the *pksG* locus.	This work
pJOE8	*amyQ* gene (P*_amyQ_*-P*_cry3A_*) knock-in plasmid derived from pJOE8999.1. Integration at the *ppsE* locus.	This work
pJOE9	*amyQ* gene (P*_amyQ_*-P*_cry3A_*) knock-in plasmid derived from pJOE8999.1. Integration at the *cotB* locus.	This work
pJOE10	*amyQ* gene (P*_amyQ_*-P*_cry3A_*) knock-in plasmid derived from pJOE8999.1. Integration at the *ylbP* locus.	This work
pJOE11	*amyQ* gene (P*_amyQ_*-P*_cry3A_*) knock-in plasmid derived from pJOE8999.1. Integration at the *veg* locus.	This work
pJOE12	*hag* gene knock-out plasmid derived from pJOE8999.1	This work
pJOE13	*pssA* gene knock-out plasmid derived from pJOE8999.1	This work
pJOE14	*yusX* gene knock-out plasmid derived from pJOE8999.1	This work
pJOE15	*hrcA* gene knock-out plasmid derived from pJOE8999.1	This work
pJOE16	*sipT* gene knock-in plasmid derived from pJOE8999.1. Integration at the *thrC* locus.	This work
pJOE17	*rasP* gene knock-in plasmid derived from pJOE8999.1. Integration at the *spoVG* locus.	This work
pJOE18	*sppA* gene knock-in plasmid derived from pJOE8999.1. Integration at the *yqeZ* locus.	This work
pJOE19	*secYEG* artificial operon knock-in plasmid derived from pJOE8999.1. Integration at the *sigX* locus.	This work
pJOE20	*gudB* gene restoration plasmid derived from pJOE8999.1	This work

### 4.6. Plasmid Curing and Genome Edition Modifications

To cure the plasmid from recombinant strains, edited colonies were passaged three times on LB agar plates (without any antibiotics) at 50 °C for 24 h. Plasmid curing yielded the optimum results when the cells were streaked for single colonies at each passage. Colonies cured of the editing plasmid were confirmed by streaking them onto LB agar plates containing kanamycin or no antibiotics; colonies cured of plasmids fail to grow at 37 °C. The identity of each genome modification in the mutant strains was verified by colony PCR using the relevant primers and Sanger sequencing (Table S1 and Figure S1).

### 4.7. Quantification of α-Amylase Activity in Shake Flasks

Overnight cultures of recombinant *B. subtilis* strains in production medium (12 g/L sucrose, 18 g/L peptone, 2 g/L (NH_4_)_2_SO_4_, 18.3 g/L K_2_HPO_4_·3H_2_O, 6 g/L KH_2_PO_4_, 1 g/L Na^+^citrate, 0.2 g/L MgSO_4_·7H_2_O, 1 g/L CaCl_2_, 0.12 g/L FeSO_4_·7H_2_O, 30 mg/L MnSO_4_·H_2_O, 12 mg/L CuSO_4_·H_2_O, and 12 mg/L ZnCl_2_) were diluted to 0.1 OD_600_ in 25 mL of production media and grown at 37 °C and 220 rpm for 48 h. The culture supernatant, which was used as the crude enzyme solution, was obtained at various intervals during cultivation by centrifugation at 8000× *g* for 20 min at 4 °C. The enzyme activity was calculated by incubating 1 µL of the supernatant with 250 µL of 0.75% (*w*/*v*) soluble starch solution (prepared in Tris-HCl buffer at a pH of 6.5) as a substrate for 1 min at 80 °C. The reaction was terminated by adding 0.75 mL of 3,5-dinitrosalicylic acid (DNS) reagent, followed by incubating the mixture in a boiling water bath for 5 min. Absorbance was read at 540 nm and compared to a standard curve. One unit (IU) of amylase activity was defined as the amount of enzyme that liberated 1 µmol of maltose from soluble starch per minute under the assay conditions.

### 4.8. SDS-PAGE Analysis

After the growth of the cells for 48 h at 37 °C and 220 rpm in production media, 8 mL of the cells was harvested by centrifugation (12,000× *g*, 10 min, 4 °C) to obtain supernatant (crude enzyme solution) and a cell pellet, which was then resuspended in 800 μL of 0.05 M Tris-HCl with a pH of 7. The crude cell extract was prepared using an ultrasonic homogenizer (Bandelin Sonoplus HD 3100, Berlin, Germany) for 1 min at 70% amplitude using the following cycle: work 1 s, stop 1 s. After centrifugation, the supernatant of the lysate and the supernatant of the cell cultures were analyzed using sodium dodecyl sulphate–polyacrylamide gel electrophoresis (SDS-PAGE) with a 10% separation gel. Proteins were visualized with Coomassie Brilliant Blue.

### 4.9. Dry Cell Weight

The dry cell weight (DCW) of the parental strain BS1 was determined by centrifuging 10 mL of BS1 culture broth at 12,000× *g* for 15 min at 4 °C. The resulting pellet was washed three times in 0.9% (*w*/*v*) NaCl solution before drying it in an oven at 105 °C to obtain a constant weight. OD_600_ was monitored using a Genesys 30 visible spectrophotometer (Thermo Fisher Scientific, Waltham, MA, USA) and used to convert the optical density at 600 nm (OD_600_) into the DCW. According to the formula, 1 OD_600_ was equivalent to 0.352 g/L. This formula was used to calculate the DCW of each recombinant strain.

### 4.10. Three-Liter Fermentor Experiments

Seed culture was started by inoculating 50 mL of the production medium into a 500 mL shake flask with a 20 μL sample of frozen glycerol stock (stored at −80 °C). The resulting cultures were incubated at 37 °C and 220 rpm for 18 h. In a 3 L fermentor (Sartorius AG, Göttingen, Germany), the fermentation medium (30 g/L sucrose, 30 g/L peptone, 2 g/L (NH_4_)_2_SO_4_, 18.3 g/L K_2_HPO_4_·3H_2_O, 6 g/L KH_2_PO_4_, 1 g/L Na^+^citrate, 0.2 g/L MgSO_4_·7H_2_O, 1 g/L CaCl_2_, 0.12 g/L FeSO_4_·7H_2_O, 30 mg/L MnSO_4_·H_2_O, 12 mg/L CuSO_4_·H_2_O, and 12 mg/L ZnCl_2_) was inoculated with seed cultures at a ratio of 4% (*v*/*v*). Fed-batch fermentation with pulse feeding was started as a 0.9 L batch and was carried out at 37 °C, maintaining the pH at 7 by using 4 M H_3_PO_4_ and 2 M NaOH and the dissolved oxygen (DO) at approximately 40% by cascading the agitation speed between the ranges of 700–1000 rpm and injecting air mixed with pure oxygen. Two pulse feeds of 0.12 L at 24 h and 48 h were added to the fermentor. The first pulse contained 250 g/L sucrose and 250 g/L soy peptone, along with other nutrients, whereas in the second pulse, the sucrose was increased to 580 g/L and was fed in when the dissolved oxygen concentration in the reactor increased abruptly, indicating complete consumption of the carbon source. At defined time intervals, the medium was sampled. The culture supernatant was obtained by centrifugation at 12,000× *g* for 15 min at 4 °C and used as the crude enzyme solution for further analysis.

### 4.11. Statistical Analysis

All the samples were analyzed in triplicate, and the data are presented as the mean ± standard deviation for each sample point. All the data were collected to analyze the variance at *p* < 0.05, and a t test was applied to compare the mean values using the R-jamovi statistical software (version 2.5).

## 5. Conclusions

Selecting a suitable promoter along with increasing the gene copy number proved to be the most important determinants to achieve the highest levels of *amyQ* expression. However, once the levels of the gene-specific message had saturated the post-transcriptional machinery, we found that the deficiency of the PrsA lipoprotein and the accumulation of signal peptides in the cytoplasmatic membrane constituted the most critical rate-limiting steps in our AmyQ protein production system, achieving a stunning 57.9-fold increase in enzyme activity and the production of AmyQ compared to the control at the fermentor stage. This high-level production provides a basis for enhanced industrial production of the α-amylase AmyQ. We believe this approach could be also valuable in the expression of other enzymes in *B. subtilis*.

## Figures and Tables

**Table 1:** Cumulative effect on AmyQ α-amylase activity of successive modifications with significant positive effects. Dry cell weight (DCW) values of the engineered strains are also shown. Experiments were carried out in triplicate, and data are presented as mean values ± standard deviation.

Strain	Amylase Activity (U/mL)	Cumulative Increase ^a^		DCW (g/L)
		Total Fold Change	*p*-Value ^b^	
BSQ1a	24.9 ± 1.5	1		4.3 ± 0.1
BSQ1b	122.8 ± 4.4	4.9	<0.001	4.7 ± 0.2
BSQ2	240.4 ± 8.0	9.7	<0.001	4.7 ± 0.1
BSQ3	315.7 ± 18.4	12.7	0.003	4.8 ± 0.2
BSQ4	415.7 ± 21.1	16.7	0.003	4.6 ± 0.1
BSQ5	501.2 ± 13.5	20.2	0.004	4.3 ± 0.4
BSQ6	520.6 ± 20.2	20.9	0.3	4.5 ± 0.2
BSQ6_7	579.0 ± 25.4	23.3	0.04	4.3 ± 0.3
BSQ6_9	604.3 ± 13.5	24.3	0.004 ^c^	4.3 ± 0.2
BSQ6_11	656.8 ± 24.3	26.4	0.03	4.4 ± 0.01
BSQ6_11_F ^d^	1439.2 ± 92.7	57.9	<0.001	22.4 ± 0.7

^a^ The increase by one significant edition after another in the total fold change. ^b^ *p*-value from Student’s *t*-test of each strain compared with the precedent strain. ^c^ *p*-value from Student’s *t*-test compared with BSQ6. ^d^ BSQ6_11 values in a 3 L fermentor at 90 h.

**Table 2:** Strains used in this study.

Strain	Characteristics	Reference
*E. coli* DH5α	*fhuA2 lac(del)U169 phoA glnV44 Φ80’ lacZ(del)M15 gyrA96 recA1 relA1 endA1 thi-1 hsdR17*	Laboratory stock
*E. coli* NEB^®^ turbo	*F’ proA + B + lacIq* ∆*lacZM15/fhuA2* ∆*(lac-proAB) glnV galK16 galE15 R(zgb-210::Tn10)TetS endA1 thi-1* ∆*(hsdS-mcrB)5*	Laboratory stock
*B. subtilis* strains		
BS0	Δ*nprE*, Δ*aprE*, Δ*epr*, Δ*mpr*, Δ*nprB*, Δ*vpr*, Δ*bpr*, Δ*sigF*	BGSC
BS1	BS0 derivative, Δ*lytC*	Laboratory stock
BS2	BS1 derivative, Δ*amyE*	This work
BSQ1a	BS2 derivative, *amyQ* (P*_spoVG_*) knock-in mutant (Δ*spoVG*)	This work
BSQ1b	BS1 derivative, *amyQ* (P*_amyQ_*-P*_cry3A_*) knock-in mutant (Δ*amyE*)	This work
BSQ1c	BS2 derivative, *amyQ* (P*_spoVG_*-P*_amyQ_*-P*_cry3A_*) knock-in mutant (Δ*spoVG*)	This work
BSQ1d	BS2 derivative, *amyQ* (SP*_ywbN’_*) knock-in mutant (Δ*ywbN*)	This work
BSQ1e	BS2 derivative, *amyQ* (P*_amyQ_*-P*_cry3A_*) knock-in mutant (Δ*pksG*)	This work
BSQ1f	BS2 derivative, *amyQ* (P*_amyQ_*-P*_cry3A_*) knock-in mutant (Δ*ppsE*)	This work
BSQ1g	BS2 derivative, *amyQ* (P*_amyQ_*-P*_cry3A_*) knock-in mutant (Δ*cotB*)	This work
BSQ1h	BS2 derivative, *amyQ* (P*_amyQ_*-P*_cry3A_*) knock-in mutant (Δ*ylbP*)	This work
BSQ1i	BS2 derivative, *amyQ* (P*_amyQ_*-P*_cry3A_*) knock-in mutant (Δ*veg*)	This work
BSQ2	BSQ1b derivative, *amyQ* double knock-in mutant (Δ*amyE*, Δ*pksG*)	This work
BSQ3	BSQ2 derivative, *amyQ* triple knock-in mutant (Δ*amyE*, Δ*pksG*,Δ*ppsE*)	This work
BSQ4	BSQ3 derivative, *amyQ* quadruple knock-in mutant (Δ*amyE*, Δ*pksG*,Δ*ppsE*, Δ*cotB*)	This work
BSQ5	BSQ4 derivative, *amyQ* quintuple knock-in mutant (Δ*amyE*, Δ*pksG*, Δ*ppsE*, Δ*cotB*, Δ*ylbP*)	This work
BSQ6	BSQ4 derivative, *amyQ* sextuple knock-in mutant (Δ*amyE*, Δ*pksG*, Δ*ppsE*, Δ*cotB*, Δ*ylbP*, Δ*veg*)	This work
BSQ6_1	BSQ6 derivative, Δ*hag*	This work
BSQ6_2	BSQ6 derivative, Δ*pssA*	This work
BSQ6_3	BSQ6 derivative, Δ*yusX*	This work
BSQ6_4	BSQ6 derivative, Δ*hrcA*	This work
BSQ6_5	BSQ6 derivative, *sipT* knock-in mutant (Δ*thrC*)	This work
BSQ6_6	BSQ6 derivative, *rasP* knock-in mutant (Δ*spoVG*)	This work
BSQ6_7	BSQ6 derivative, *sppA* knock-in mutant (Δ*yqeZ*)	This work
BSQ6_8	BSQ6 derivative, *secYEG* knock-in mutant (Δ*sigX*)	This work
BSQ6_9	BSQ6 derivative, *prsA* knock-in mutant (Δ*pel*; erm^r^)	This work
BSQ6_10	BSQ6 derivative, restored *gudB* gene	This work
BSQ6_11	BSQ6 derivative, *sppA* and *prsA* knock-in mutant (Δ*yqeZ*, Δ*pel*, erm^r^)	This work

BGSC: Bacillus Genetic Stock Center.

## Data Availability

All the data supporting the conclusions of this study are included within the article and its Supplementary Materials.

## Supplementary Materials

The following supporting information can be downloaded at https://www.mdpi.com/article/10.3390/ijms25136957/s1.
