# Supplementary material for: The Construction of an Environmentally Friendly Super-Secreting Strain of Bacillus subtilis through Systematic Modulation of Its Secretory Pathway Using the CRISPR-Cas9 System
Source: Int J Mol Sci. 2024 Jun 25;25(13):6957. doi: 10.3390/ijms25136957 (PMC11240994; doi:10.3390/ijms25136957)
Supplement: Supplementary file 1 [file ijms-25-06957-s001.zip › ijms-3038174-supplementary.pdf]

Construction of an environmentally friendly super-secreting strain of *Bacillus subtilis* by systematic modulation of its secretory pathway using the CRISPR-Cas9 system

Jordi Ferrando, David Miñana-Galbis and Pere Picart

SUPPLEMENTARY MATERIAL

Additional file 1

Figure S1. PCR verification of *B. subtilis* mutants

Figure S2. *amyQ* gene and dual promoter (P_{amyQ}-P_{cry3A}) sequence with codon optimization for *B. subtilis*

Figure S3. Sanger sequencing results of *gudB* gene in BSQ6_10 strain

Figure S4. Sequence of the synthetic *prsA* gene used in this study

Table S1. Primers designed in this study

Table S2. Splicing with overlap extension PCR (SOEing-PCR) program

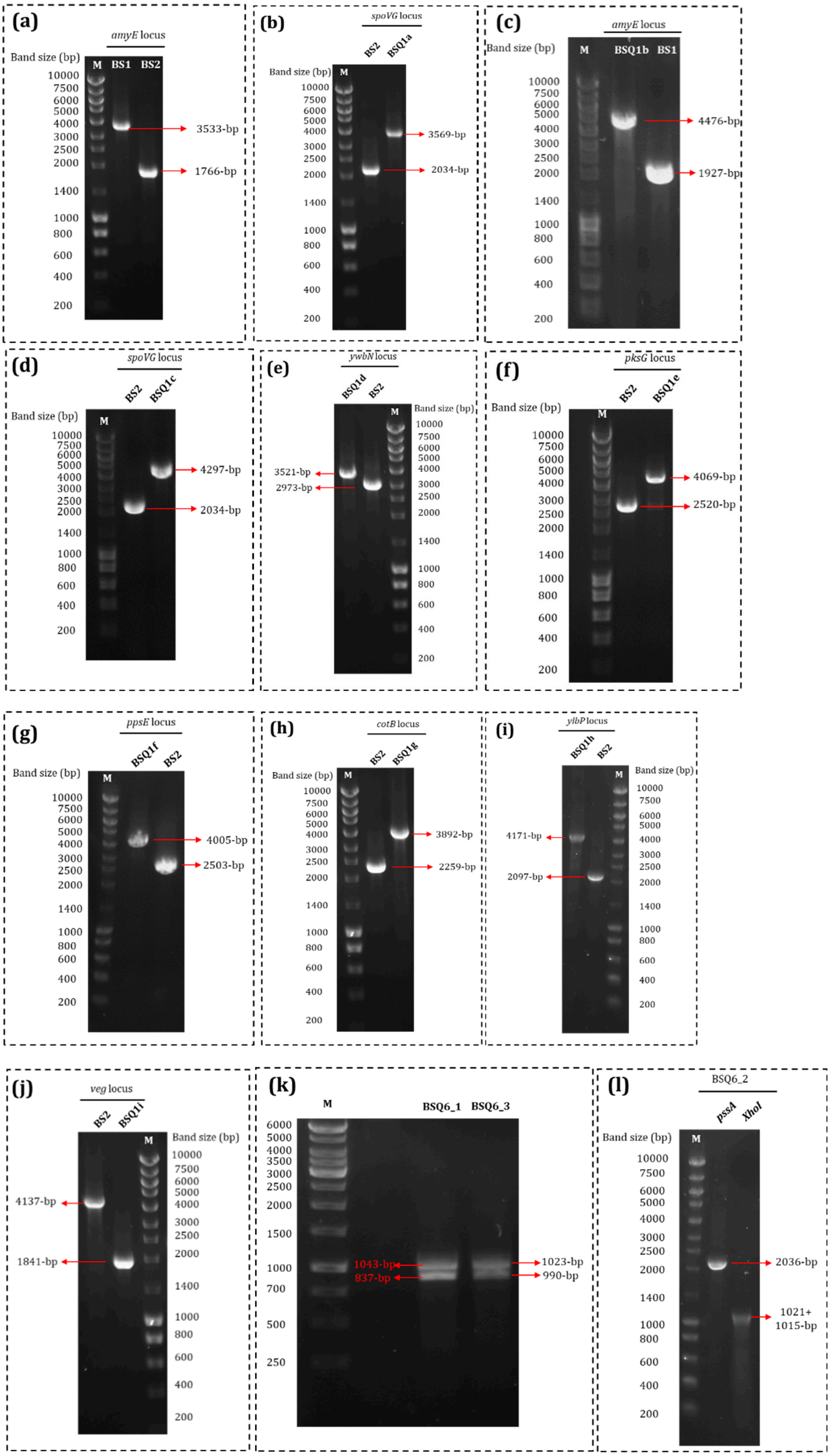

(a)
amyE locus
Band size (bp)
M BS1 BS2
10000
7500
6000
5000
4000
3000
2500
2000
1400
1000
800
600
400
200
3533-bp
1766-bp
(b)
spoVG locus
BS2 BSQ1a
Band size (bp) M
10000
7500
6000
5000
4000
3000
2500
2000
1400
1000
800
600
400
200
3569-bp
2034-bp
(c)
amyE locus
Band size (bp)
M BSQ1b BS1
10000
7500
6000
5000
4000
3000
2500
2000
1400
1000
800
600
400
200
4476-bp
1927-bp
(d)
spoVG locus
BS2 BSQ1c
Band size (bp)
M
10000
7500
6000
5000
4000
3000
2500
2000
1400
1000
800
600
400
200
4297-bp
2034-bp
(e)
ywbN locus
BSQ1d BS2
Band size (bp)
M
10000
7500
6000
5000
4000
3000
2500
2000
1400
1000
800
600
400
200
3521-bp
2973-bp
(f)
pksG locus
BS2 BSQ1e
Band size (bp)
M
10000
7500
6000
5000
4000
3000
2500
2000
1400
1000
800
600
400
200
4069-bp
2520-bp
(g)
ppsE locus
BSQ1f BS2
Band size (bp)
M
10000
7500
6000
5000
4000
3000
2500
2000
1400
1000
800
600
400
200
4005-bp
2503-bp
(h)
cotB locus
BS2 BSQ1g
Band size (bp) M
10000
7500
6000
5000
4000
3000
2500
2000
1400
1000
800
600
400
200
3892-bp
2259-bp
(i)
ylbP locus
BSQ1h BS2
M
Band size (bp)
10000
7500
6000
5000
4000
3000
2500
2000
1400
1000
800
600
400
200
4171-bp
2097-bp
(j)
veg locus
BS2 BSQ1i
M
Band size (bp)
10000
7500
6000
5000
4000
3000
2500
2000
1400
1000
800
600
400
200
4137-bp
1841-bp
(k)
M
BSQ6_1 BSQ6_3
6000
5000
4000
3500
3000
2500
2000
1500
1000
700
500
250
1043-bp
837-bp
1023-bp
990-bp
(l)
BSQ6_2
pssA XhoI
Band size (bp)
M
10000
7500
6000
5000
4000
3000
2500
2000
1400
1000
800
600
400
200
2036-bp
1021+
1015-bp

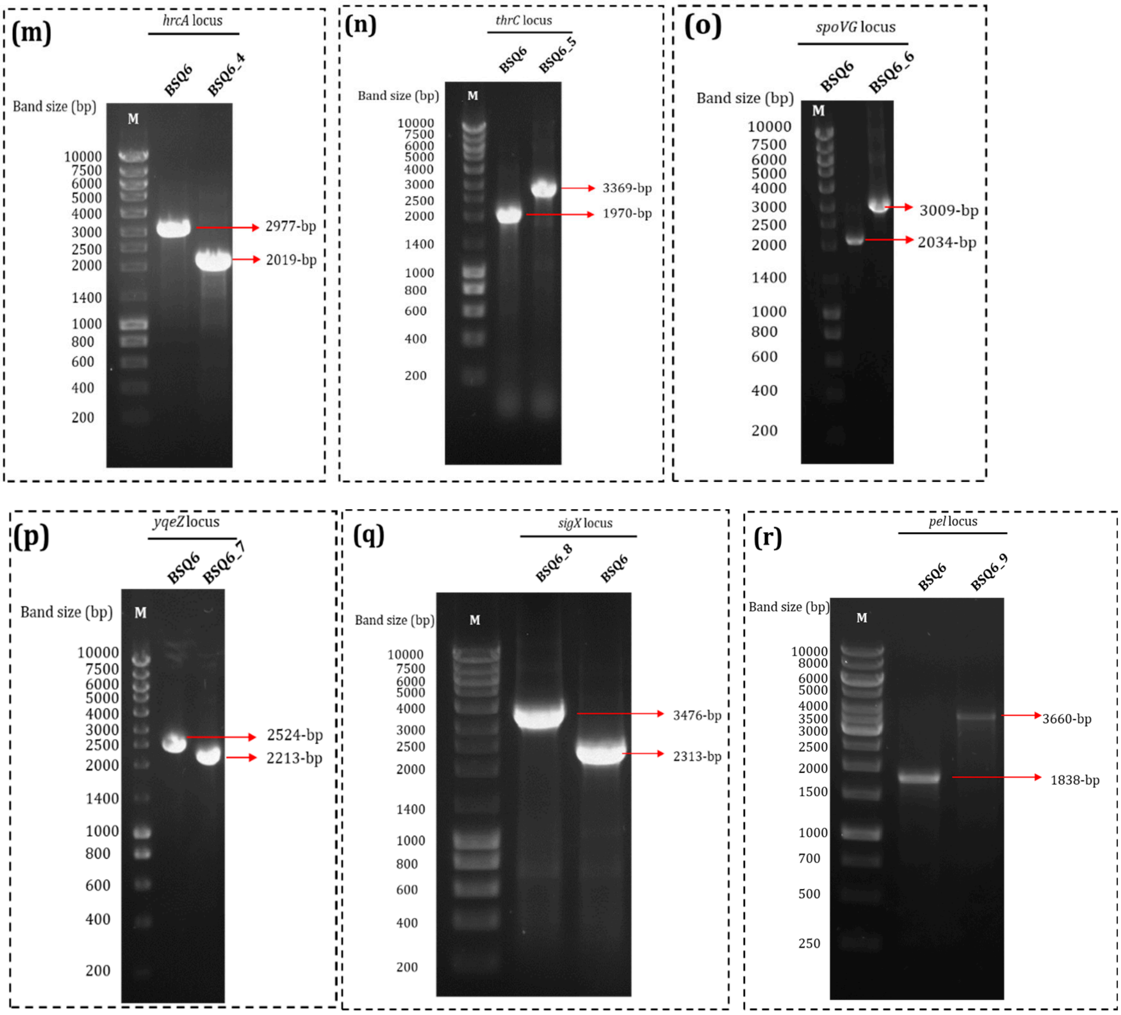

Figure S1. PCR verification of *B. subtilis* mutants. **(a)** *amyE* deletion in BS2 strain. BS1 strain was used as control; **(b)** P_{spoVG}-*amyQ* integration in BSQ1a. BS2 strain was used as control; **(c)** P_{amyQ}-P_{cry3A}-*amyQ* (*amyQ*_Ec) integration in BSQ1b strain. BS1 strain was used as control; **(d)** P_{spoVG}-P_{amyQ}-P_{cry3A}-*amyQ* integration in BSQ1c strain. BS2 strain was used as control; **(e)** *amyQ* gene with $SP_{ywbN'}$ integration in BSQ1d. BS2 strain was used as control; **(f)** *amyQ*_Ec integration in BSQ1e. BS2 strain was used as control; **(g)** *amyQ*_Ec integration in BSQ1f. BS2 strain was used as control; **(h)** *amyQ*_Ec integration in BSQ1g. BS2 strain was used as control; **(i)** *amyQ*_Ec integration in BSQ1h. BS2 strain was used as control; **(j)** *amyQ*_Ec integration in BSQ1i. BS2 strain was used as control; **(k)** insertion of Stop codon within *hag* and *yusX* genes in BSQ6_1 and BSQ6_3, respectively. PCR product digested with *XhoI*; **(l)** insertion of Stop codon within *pssA* gene in BSQ1_2. pssA, PCR product. XhoI, PCR product after digestion with *XhoI*; **(m)** *hrcA* deletion in BSQ6_4. BSQ6 strain was used as control; **(n)** *sipT* integration in BSQ6_5. BSQ6 strain was used as control; **(o)** *rasP* integration in BSQ6_6. BSQ6 strain was used as control; **(p)** *sppA* integration in BSQ6_7. BSQ6 strain was used as control; **(q)** *secYEG* operon integration in BSQ6_8. BSQ6 strain was used as control; **(r)** *prsA* integration in BSQ6_9. BSQ6 strain was used as control. M corresponds to the molecular weight marker. For knock-out strains, the following corresponding verification primer pairs were used: P1_1F/P1_2R (for *lytC*), P2_1F/P2_2R (for *amyE*), P12_1F/P12_2R (for *hag*), P13_1F/P13_2R (for *pssA*), P14_1F/P14_2R (for *yusX*) and P15_1F/P15_2R (for *hrcA*); whereas for knock-in strains the following primers were used: P5_1F/P5_3R (for *spoVG* site), P4_1F/P4_3R (for *amyE* site), P6_1F/P6_3R (for *ywbN* site), P7_1F/P7_3R (for *pksG* site), P8_1F/P8_3R (for *ppsE* site), P9_1F/P9_3R (for *cotB* site), P10_1F/P10_3R (for *ylbP* site), P11_1F/P11_3R (for *veg* site), P16_1F/P16_3R (for *thrC* site), P18_1F/P18_3R (for *yqeZ* site), P19_1F/P19_5R (for *sigX* site), P20_1F/P20_2R (for *gudB* site) and pelF/pelR (for *pel* site).

TGCTGTCCAGACTGTCCGCTGTGTAAAAATAAGGAATAAAGGGGGGTTGACATTATTTTACTGATATGTATAATATAA
TTTGTATAAGAAAATGTCGAAACGTAAGATGAAACCTTAGATAAAAGTGCTTTTTTTGTTGCAATTGAAGAATTATTA
ATGTTAAGCTTAATTAAAGATAATATCTTTGAATTGTAACGCCCCTCAAAAGTAAGAACTACAAAAAAAGAATACGTT
ATATAGAAATATGTTTGAACCTTCTTCAGATTACAAATATATTCGGACGGACTCTACCTCAAATGCTTATCTAACTAT
AGAATGACATACAAGCACAACCTTGAAAATTTGAAAATATAACTACCAATGAACTTGTTCATGTGAATTATCGCTGTA
TTTAATTTTCTCAATTCAATATATAATATGCCAATACATTGTTACAAGTAGAAATTAAGACACCCTTGATAGCCTTAC
TATACCTAACATGATGTAGTATTAAATGAATATGTAAATATATTTATGATAAGAAGCGACTTATTTATAATCATTACA
TATTTTTCTATTGGAATGATTAAGATTCCAATAGAATAGTGTATAAATTATTTATCTTGAAAGGAGGGATGCCTAAAA
ACGAAGAACATTAAAAACATATATTTGCACCGTCTAATGGATTTATGAAAAATCATTTTATCAGTTTGAAAATTATGT
ATTATGATAAGAAAGGGAGGACAAAC**ATGATTCAAAAACGAAAGCGGACAGTTTCGTTCAGACTTGTGCTTATGTGCA**
CGCTGTTATTTGTCAGTTTGCCGATTACAAAAACATCAGCCGTTAACGGCACACTTATGCAATACTTCGAATGGTACA
CACCTAACGATGGCCAACATTGGAAACGTCTTCAAAACGATGCTGAACATCTTTCTGATATCGGCATCACAGCTGTTT
GGATACCTCCTGCTTACAAAGGCCTTTCTCAATCTGATAACGGCTACGGCCCTTACGATCTTTACGATCTTGGCGAAT
TCCAACAAAAAGGCACAGTTCGTACAAAATACGGCACAAAATCTGAACTTCAAGATGCTATCGGCTCTCTTCATTCTC
GTAACGTTCAAGTTTACGGCGATGTTGTTCTTAACCATAAAGCTGGCGCTGATGCTACAGAAGATGTTACAGCTGTTG
AAGTTAACCCTGCTAACCGTAACCAAGAAACATCTGAAGAATACCAAATCAAAGCTTGGACAGATTTCCGTTTCCCTG
GCCGTGGCAACACATACTCTGATTTCAAATGGCATTGGTACCATTTCGATGGCGCTGATTGGGATGAATCTCGTAAAA
TCTCTCGTATCTTCAAATTCCGTGGCGAAGGCAAAGCTTGGGATTGGGAAGTTTCTTCTGAAAACGGCAACTACGATT
ACCTTATGTACGTTGATGTTGATTACGATCATCCTGATGTTGTTGCTGAAACAAAAAAATGGGGCATCTGGTACGCTA
ACGAACTTTCTCTTGATGGCTTCCGTATCGATGCTGCTAAACATATCAAATTCTCTTTCCTTCGTGATTGGGTTCAAG
CTGTTCGTCAAGCTACAGGCAAAGAAATGTTCACAGTTGCTGAATACTGGCAAAACGATGCTGGCAAACTTGAAAACT
ACCTTAACAAAACATCTTTCAACCAATCTGTTTTCGATGTTCCTCTTCATTTCAACCTTCAAGCTGCTTCTTCTCAAG
GCGGCGGCTACGATATGCGTCGTCTTCTTGATGGCACAGTTGTTTCTCGTCATCCTGAAAAAGCTGTTACATTCGTTG
AAAACCATGATACACAACCTGGCCAATCTCTTGAATCTACAGTTCAAACATGGTTCAAACCTCTTGCTTACGCTTTCA
TCCTTACACGTGAATCTGGCTACCCTCAAGTTTTCTACGGCGATATGTACGGCACAAAAGGCACATCTCCTCGTGAAA
TCCCTTCTCTTAAAGATTCTATCGAACCTATCCTTAAAGCTCGTAAAGAATACGCTTACGGCCCTCAACATGATTACA
TCGATCATCCTGATGTTATCGGCTGGACACGTGAAGGCGATTCTTCTGCTGCTAAATCTGGCCTTGCTGCTCTTATCA
CAGATGGCCCTGGCGGCTCTAAACGTATGTACGCTGGCCTTAAAAACGCTGGCGAAACATGGTACGATATCACAGGCA
ACCGTTCTGATACAGTTAAAATCGGCTCTGATGGCTGGGGCGAATTCCATGTTAACGATGGCTCTGTTTCTATCTACG
TTCAAAAATAA*GGTAATAAAAAAACACCTCCAAGCTGAGTGCGGGTATCAGCTTGGAGGTGCGTTTATTTTTTCAGCC*
GTATGACAAGGTCGGCATCAGGTGTGACAAATACGGTATGCTGGCTGTCATAGGTGACAAATCCGGGTTTTGCGCCGT
TTGGCTTTTTCACATGTCTGATTTTTGTATAATCAACAGGCACGGAGCCGGAATCTTTCGCCTTGGAAAAATAAGCGG
CGATCGTAGCTGCTTCCAATATGGATTGTTCATCGGGATCGCTGCTTTTAATCACAACGTG

Figure S2. *amyQ* gene and dual promoter (P_{amyQ}-P_{cry3A}) sequence with codon optimization for *B. subtilis*. The signal peptide SP_{amyQ} is marked in bold. Promoter and terminator are underlined.

Cryptic *gudB* gene in *B. subtilis* BS1 strain due to a duplication of 9 bp:

ATGGCAGCCGATCGAAACACCGGTCATACAGAAGAGGACAAACTTGATGTATTAAAATCAACCCAAACCGTAATACATAAGGCT
CTGGAAAAATTGGGATATCCCGAAGAGGTATACGAATTGTTAAAAGAGCCGATGAGATTATTAACGGTAAAAATACCTGTTCGT
ATGGACGACGGTTCAGTAAAGATTTTCACAGGATATCGTGCGCAGCACAATGACTCTGTCGGTCCAACGAAAGGCGGGATACG
TTTTCACCCGAACGTAACAGAAAAAGAG**GTGAAGGCGGTGAAGGCG**CTTTCAATTTGGATGAGTTTAAAATGCGGCATAATTG
ATCTTCCATATGGCGGTGGTAAAGGCGGAATTGTTTGTGATCCAAGGGATATGTCGTTTAGAGAGCTGGAGCGTCTGAGCAGA
GGGTATGTCAGAGCGATCAGCCAAATTGTCGGCCCGACAAAAGACGTGCCGGCACCGGATGTATTTACAAACTCACAAATCAT
GGCTTGGATGATGGATGAGTATTCAAGAATTGATGAATTTAATTCGCCTGGATTTATTACAGGCAAACCGCTTGTGCTTGGCGG
ATCTCACGGGAGAGAATCTGCGACAGCAAAAGGTGTTACCATCTGTATTAAAGAAGCGGCTAAGAAGAGAGGCATCGATATTA
AAGGTGCGCGTGTCGTTGTCCAAGGCTTCGGAAACGCGGGAAGCTATTTGGCAAAATTTATGCATGATGCGGGGGCAAAAGTT
GTCGGCATCTCAGATGCGTATGGCGGACTTTATGATCCGGAAGGCCTTGATATCGATTATTTACTCGACCGACGCGACAGCTTC
GGTACCGTAACAAAGCTTTTCAACGATACCATTACCAACCAAGAGCTGCTGGAGCTGGATTGTGATATTCTCGTTCCTGCTGCGA
TTGAAAATCAAATTACAGAAGAAAATGCCCATAATATCCGGGCTAAAATTGTCGTTGAAGCAGCGAACGGACCAACAACGCTTG
AAGGAACAAAAATTCTTTCAGACCGGGACATTCTGCTTGTACCAGACGTGCTGGCAAGTGCCGGTGGCGTAACAGTTTCTTATT
TTGAATGGGTTCAGAATAACCAAGGCTTCTACTGGAGTGAAGAAGAGGTAGAAGAAAAATTAGAAAAAATGATGGTCAAATCA
TTTAACAATATTTACGAAATGGCTAACAACCGAAGAATTGACATGAGGCTCGCTGCATATATGGTCGGCGTTCGCAAAATGGCT
GAAGCTTCGCGTTTTAGAGGCTGGATATAA

Sequencing results of restored *gudB* gene in BSQ6_10 strain with removed 9-bp direct repeat:

ATGGCAGCCGATCGAAACACCGGTCATACAGAAGAGGACAAACTTGATGTATTAAAATCAACCCAAACCGTAATACATAAGGCT
CTGGAAAAATTGGGATATCCCGAAGAGGTATACGAATTGTTAAAAGAGCCGATGAGATTATTAACGGTAAAAATACCTGTTCGT
ATGGACGACGGTTCAGTAAAGATTTTCACAGGATATCGTGCGCAGCACAATGACTCTGTCGGTCCAACGAAAGGCGGGATACG
TTTTCACCCGAACGTAACAGAAAAAGAG**GTGAAGGCG**CTTTCAATTTGGATGAGTTTAAAATGCGGCATAATTGATCTTCCATA
TGGCGGTGGTAAAGGCGGAATTGTTTGTGATCCAAGGGATATGTCGTTTAGAGAGCTGGAGCGTCTGAGCAGAGGGTATGTCA
GAGCGATCAGCCAAATTGTCGGCCCGACAAAAGACGTGCCGGCACCGGATGTATTTACAAACTCACAAATCATGGCTTGGATG
ATGGATGAGTATTCAAGAATTGATGAATTTAATTCGCCTGGATTTATTACAGGCAAACCGCTTGTGCTTGGCGGATCTCACGGG
AGAGAATCTGCGACAGCAAAAGGTGTTACCATCTGTATTAAAGAAGCGGCTAAGAAGAGAGGCATCGATATTAAAGGTGCGCG
TGTCGTTGTCCAAGGCTTCGGAAACGCGGGAAGCTATTTGGCAAAATTTATGCATGATGCGGGGGCAAAAGTTGTCGGCATCTC
AGATGCGTATGGCGGACTTTATGATCCGGAAGGCCTTGATATCGATTATTTACTCGACCGACGCGACAGCTTCGGTACCGTAAC
AAAGCTTTTCAACGATACCATTACCAACCAAGAGCTGCTGGAGCTGGATTGTGATATTCTCGTTCCTGCTGCGATTGAAAATCAA
ATTACAGAAGAAAATGCCCATAATATCCGGGCTAAAATTGTCGTTGAAGCAGCGAACGGACCAACAACGCTTGAAGGAACAAA
AATTCTTTCAGACCGGGACATTCTGCTTGTACCAGACGTGCTGGCAAGTGCCGGTGGCGTAACAGTTTCTTATTTTGAATGGGTT
CAGAATAACCAAGGCTTCTACTGGAGTGAAGAAGAGGTAGAAGAAAAATTAGAAAAAATGATGGTCAAATCATTTAACAATAT
TTACGAAATGGCTAACAACCGAAGAATTGACATGAGGCTCGCTGCATATATGGTCGGCGTTCGCAAAATGGCTGAAGCTTCGC
GTTTTAGAGGCTGGATATAA

Figure S3. Sanger sequencing results of *gudB* gene in BSQ6_10 strain. The 9-bp repeat (GTGAAGGCG) is marked in bold.

AATAGCCAACATTGACATTATTTTACTGATATGTATAATATAATTTGTATAAGAAAATGAGAGGGAGAGG
AAACATGAAGAAGATTGCAATTGCGGCGATTACAGCGACAAGCGTGCTGGCTCTCAGCGCATGCTCTGGC
GGCGATTCTGAAGTTGTTGCTGAAACAAAAGCTGGCAACATCACAAAAGAAGATCTTTACCAAACACTTA
AAGATAACGCTGGCGCTGATGCTCTTAACATGCTTGTTCAAAAAAAAGTTCTTGATGATAAATACGATGT
TTCTGATAAAGAAATCGATAAAAAACTTAACGAATACAAAAAATCTATGGGCGATCAACTTGATCAACTT
ATCAAACAAAAAGGCGAAGATTTCGTTAAAGAACAAATCAAATACGAACTTCTTATGAAAAAAGCTGCTA
AAGATAACATCAAAGTTACAGATGATGATGTTAAAGAATACTACGATGGCCTTAAAGGCAAAATCCATCT
TTCTCATATCCTTGTTAAAGAAAAAAAAACAGCTGAAGAAGTTGAAAAAAAACTTAAAAAAGGCGAAAAA
TTCGAAGATCTTGCTAAAGAATACTCTACAGATGGCACAGCTGAAAAAGGCGGCGATCTTGGCTGGGTTG
GCAAAGATGATAACATGGATAAAGATTTCGTTAAAGCTGCTTTCGCTCTTAAAACAGGCGAAATCTCTGG
CCCTGTTAAATCTCAATTCGGCTACCATATCATCAAAAAAGATGAAGAACGTGGCAAATACGAAGATATG
AAAAAAGAACTTAAAAAAGAAGTTGAAGAACAAAAACTTAACGATCAAACAGAACTTCAATCTGTTATCG
ATAAACTTGTTAAAGATGCTGATCTTAAAGTTAAAGATAAAGAACTTAAAAAACAAGTTGATCAACGTCA
AGCTCAAACATCTTCTTCTTCTTAA*CCAGGCATCAAATAAAACGAAAGGCTCAGTCGAAAGACTGGGCCT*
TTCGTTTTATCTGTTGTTTGTCGGTGAACGCTCTCTACTAGAGTCACACTGGCTCACCTTCGGGTGGGCC
TTTCTGCGTTTATA

Figure S4. Sequence of synthetic *prsA* gene used in this study. Promoter is underlined and terminator is in italics

Name	Sequence	Purpose	PCR Product
TS1 F	5' – TACGAAAAATGTAATTATTGTAGG	**Target sequence for *lytC* gene deletion**	
TS1 R	5' – AAACCCTACAATAATTACATTTTT		
		**PCR of homology template for *lytC* gene deletion**	**2101**
P1_1F	5' – AAGGCCAACGAGGCCGAACTACTGTACCTGATACAAC	Upstream *lytC* gene homologous arm	1108
P1_1R	5' – GGTGTTCCCATGCCTCGAGAATAATTACATTTTTAGTCTGCATC		
P1_2F	5' – CTCGAGGCATGGGAACACCTGCTGTTTCTTC	Downstream *lytC* gene homologous arm	993
P1_2R	5' – AAGGCCTTATTGGCCTCTCTCGTTCCAAGATTAGCC		
TS2 F	5' – TACGACAGCACCGTCGATCAAAAG	**Target sequence for *amyE* gene deletion**	
TS2 R	5' – AACCTTTTGATCGACGGTGCTGT		
		**PCR of homology template for *amyE* gene deletion**	**1766**
P2_1F	5' – AAGGCCAACGAGGCCGAGTTATTCATTGCAGAAGCGCA	Upstream *amyE* gene homologous arm	890
P2_1R	5' – GTTGTATCCCCTGCCAGAACCAAATGAAAC		
P2_2F	5' – GTTCTGGCAGGGGATACAACCAACGCAAAAGTG	Downstream *amyE* gene homologous arm	876
P2_2R	5' –AAGGCCTTATTGGCCGTGGCTCCAAACAGGAAGC		
TS3 F	5' – TACGAAATCAGACATGCTCGGACG	**Target sequence for *spoVG* replacement**	
TS3 R	5' – AAACCGTCCGAGCATGTCTGATTT		
		**PCR of homology template for *spoVG* gene replacement for *amyQ gene* (single promoter)**	**2894**
P3_1F	5' – AAGGCCAACGAGGCCCGGGCCTTATTCACAAGGG	Upstream *spoVG* gene homologous arm	530
P3_1R	5' – CTTTCGTTTTTGAATCATAGTAGTTCACCACCTTTTCCC		
P3_2F	5' – GTGAACTACTATGATTCAAAAACGAAAGCGGAC	*amyQ* gene amplification	1829
P3_2R	5' – GGTTATTTTCACGTTGTGATTAAAAGCAGCG		
P3_3F	5' – GCTTTTAATCACAACGTGAAAATAACCAAAAAGCAAGGACTG	Downstream *spoVG* gene homologous arm	535
P3_3R	5' – AAGGCCTTATTGGCCTTACTGTTCCATCGTCTCTGCTG		
		**PCR of homology template for *amyQ* gene integration at *amyE* site**	**4476**
P4_1F	5' – AAGGCCAACGAGGCCGAGTTATTCATTGCAGAAGCGCA	Upstream *amyE* gene homologous arm	951
P4_1R	5' – GCGGACAGTCTGGACAGCAGATCGACGGTGCTGTAAGCT		
P4_2F	5' – AGCTTACAGCACCGTCGATCTGCTGTCCAGACTGTCCGC	*amyQ* gene amplification	2557
P4_2R	5' – CCATGCATGAAGAATGGTTCCTCACGTTGTGATTAAAAGCAGCGAT		
P4_3F	5' – ATCGCTGCTTTTAATCACAACGTGAGGAACCATTCTTCATGCATGG	Downstream *amyE* gene homologous arm	968
P4_3R	5' – AAGGCCTTATTGGCCCTTCAAATAAAGCACTCCCGC		
		**PCR of homology template for *spoVG* gene replacement for *amyQ gene* (triple promoter)**	**4297**
P5_1F	5' – AAGGCCAACGAGGCCCTTGCGAAAAGAAGCATGAAAAC	Upstream *spoVG* gene homologous arm	864
P5_1R	5' – CCCTATATAAAAGCATTAGTGTATC		
P5_2F	5' – ACACTAATGCTTTTATATAGGGTGCTGTCCAGACTGTCCG	*amyQ* gene amplification	2557
P5_2R	5' – GGTTATTTTCACGTTGTGATTAAAAGCAGCG		
P5_3F	5' – GCTTTTAATCACAACGTGAAAATAACCAAAAAGCAAGGACTG	Downstream *spoVG* gene homologous arm	876
P5_3R	5' – AAGGCCTTATTGGCCATTAACTCCGAGCGTTTCTTGG		
TS6 F	5' – TACGTTTAAAATGGGGAGCGATGG	**Target sequence for *ywbN* replacement**	

**TS6R**	5′ – AAACCCATCGCTCCCCATTTTAAA		
		**PCR of homology template for *ywbN* gene replacement for *amyQ gene* (Tat pathway)**	**3521**
**P6_1F**	5′ – AAGGCCAACGAGGCCCTCAGTGGAAAAAGGAGACC	Upstream *ywbN* gene homologous arm	1062
**P6_1R**	5′ – GATTTTGAATTGTCCCGTCTGTGAATTTGTTTTGGCTTTTTTCTCTGTTTATCGCTCATGATGTTACAAAACT		
**P6_2F**	5′ – CACAGACGGGACAATTCAAAATCGGGAGCGATGGCAGGGGCAGCCGTTGCGGTTAACGGCACACTTATGCAAT	*amyQ* gene amplification	1736
**P6_2R**	5′ – GCATGCGTATAAAGCAGAACCCACGTTGTGATTAAAAGCAGC		
**P6_3F**	5′ – GGTTCTGCTTTATACGCATGC	Downstream *ywbN* gene homologous arm	723
**P6_3R**	5′ – AAGGCCTTATTGGCCGTTTCCAGTGCTGATTTGAATG		

Table S1. Primers designed in this study.

Name	Sequence	Purpose	PCR Product
**TS7F**	5’ – TACGGAGGTGTTTCAAATAGATCC	**Target sequence of *pksG* gene for *amyQ* gene integration**	
**TS7R**	5’ – AAACGGATCTATTTGAAACACCTC		
		**PCR of homology template for *amyQ* gene integration at *pksG* site**	**4069**
**P7_1F**	5’ – AAGGCCAACGAGGCCCTTGGTGCTTTAATGGATCTTTC	Upstream *pksG* gene homologous arm	
**P7_1R**	5’ – CAGAGCAAGTGATCAGCAATTC		928
**P7_2F**	5’ – GAATTGCTGATCACTTGCTCTGTGCTGTCCAGACTGTCCG	*amyQ* gene amplification	2557
**P7_2R**	5’ – CGTAGGAAAAACAGCCAATTCGCACGTTGTGATTAAAAGCAGCG		
**P7_3F**	5’ – CGAATTGGCTGTTTTTCCTACG	Downstream *pksG* gene homologous arm	884
**P7_3R**	5’ – AAGGCCTTATTGGCCCGCAAACGCAATAAATGCTTTC		
**TS8F**	5’ – TACGACCGTCAGAAATCCCAGGCG	**Target sequence of *ppsE* gene for *amyQ* gene integration**	
**TS8R**	5’ – AAACCGCCTGGGATTTCTGACGGT		
		**PCR of homology template for *amyQ* gene integration at *ppsE* site**	**4005**
**P8_1F**	5’ – AAGGCCAACGAGGCCGATTAAACTGAACGCAGACTTGC	Upstream *ppsE* gene homologous arm	686
**P8_1R**	5' – CTAGAAGAGCGACTGCTCAACATAC		
**P8_2F**	5' – GTTGAGCAGTCGCTCTTCTAGTGCTGTCCAGACTGTCCG	*amyQ* gene amplification	2557
**P8_2R**	5' – CAGGAGCAACGACAAGATTAAAGCACGTTGTGATTAAAAGCAGCG		
**P8_3F**	5' – CTTTAATCTTGTCGTTGCTCCTG	Downstream *ppsE* gene homologous arm	762
**P8_3R**	5’ – AAGGCCTTATTGGCCGACGTGTAATTGCTGTTTCTGAC		
**TS9F**	5’ – TACGCACTATAGTATAATGGCCGT	**Target sequence of *cotB* gene for *amyQ* gene integration**	
**TS9R**	5’ – AAACACGGCCATTATACTATAGTG		
		**PCR of homology template for *amyQ* gene integration at *cotB* site**	**3892**
**P9_1F**	5' – AAGGCCAACGAGGCCCACAAGAGGAACTTTGGAAGG	Upstream *cotB* gene homologous arm	670
**P9_1R**	5' – CATCAAGATCACCAGGCTATTC		
**P9_2F**	5' – GAATAGCCTGGTGATCTTGATGTGCTGTCCAGACTGTCCG	*amyQ* gene amplification	2557
**P9_2R**	5' – GTTACTGTATATCGTGGAGGTCCACGTTGTGATTAAAAGCAGCG		
**P9_3F**	5' – GACCTCCACGATATACAGTAAC	Downstream *cotB* gene homologous arm	665
**P9_3R**	5' – AAGGCCTTATTGGCCGCTGGTATTGTATTCACCTCG		
**TS10F**	5' –TACGGCTTATCAACTATAAAACGC	**Target sequence of *ylbP* gene for *amyQ* gene integration**	
**TS10R**	5' –AAACGCGTTTTATAGTTGATAAGC		
		**PCR of homology template for *amyQ* gene integration at *ylbP* site**	**4171**
**P10_1F**	5' – AAGGCCAACGAGGCCGATAAGCTGGCGTTGTCAG	Upstream *ylbP* gene homologous arm	780
**P10_1R**	5' – ACAAATCTCCCCCTTTGTTG		
**P10_2F**	5' – CAACAAAGGGGGAGATTTGTCACGTTGTGATTAAAAGCAGC	*amyQ* gene amplification	2557
**P10_2R**	5' – AATAGGCCGGTTGTTTTGATTTTGCTGTCCAGACTGTCCG		
**P10_3F**	5' – AAATCAAAACAACCGGCCTATT	Downstream *ylbP* gene homologous arm	834
**P10_3R**	5' – AAGGCCTTATTGGCCAAGCATGCGGATCATACAAC		
**TS11F**	5' –TACGCTGACGTTAAAAGCAAACGG	**Target sequence of *veg* gene for *amyQ* gene integration**	
**TS11R**	5' –AAACCCGTTTGCTTTTAACGTCAG		
		**PCR of homology template for *amyQ* gene integration at *veg* site**	**4137**
**P11_1F**	5' – AAGGCCAACGAGGCCGCCAATATCAGCATCAGGAG	Upstream *veg* gene homologous arm	797
**P11_1R**	5' – TGCATCCACCTCACTACATTT		
**P11_2F**	5' – AAATGTAGTGAGGTGGATGCACACGTTGTGATTAAAAGCAGC	*amyQ* gene amplification	2557
**P11_2R**	5' – CAAAAGGTTCACTGCCCGTTATGCTGTCCAGACTGTCCG		
**P11_3F**	5' – TAACGGGCAGTGAACCTTTTG	Downstream *veg* gene homologous arm	783
**P11_3R**	5' –AAGGCCTTATTGGCCAGCCACCGGAATTACCTTC		

Name	Sequence	Purpose	PCR Product
**TS12F**	5′ – TACGAGGTCTTCGCATCAACCGTG	**Target sequence for *hag* gene deletion**	
**TS12R**	5′ – AAACCACGGTTGATGCGAAGACCT		
		**PCR of homology template for *hag* gene deletion**	**1880**
**P12_1F**	5′ – AAGGCCAACGAGGCCGCGGGATTCCAGGCTTTTTAG	Upstream *hag* gene homologous arm	837
**P12_1R**	5′ – CATCTCCCCTAAGATCTCGAGTTGATGCGAAGACCTGAAGAAAG		
**P12_2F**	5′ – CTCGAGATCTTAGGGGAGATGACGCAGCAGGT	Downstream *hag* gene homologous arm	1043
**P12_2R**	5′ – AAGGCCTTATTGGCCGTTCAATTGATCATCCCCTATGC		
**TS13F**	5′ – TACGACGATAGGAAACTTCATTTG	**Target sequence for *pssA* gene deletion**	
**TS13R**	5′ – AAACCAAATGAAGTTTCCTATCGT		
		**PCR of homology template for *pssA* gene deletion**	**2036**
**P13_1F**	5′ – AAGGCCAACGAGGCCCTATGTTTCTACTCG	Upstream *pssA* gene homologous arm	1021
**P13_1R**	5′ – AGCAATCCCTATCGCTCGAGGAAGTTTCCT		
**P13_2F**	5′ – CTCGAGCGATAGGGATTGCTGGCGATTCAT	Downstream *pssA* gene homologous arm	1015
**P13_2R**	5′ – AAGGCCTTATTGGCCGATTCGTCAGCTCTA		
**TS14F**	5′ – TACGCTTGAAACCTATAAAGCGCG	**Target sequence for *yusX* gene deletion**	
**TS14R**	5′ – AAACCGCGCTTTATAGGTTTCAAG		
		**PCR of homology template for *yusX* gene deletion**	**2013**
**P14_1F**	5′ – AAGGCCAACGAGGCCTTCCGCTTTTTCAGGCTGTC	Upstream *yusX* gene homologous arm	990
**P14_1R**	5′ – TCCCAGCCCAGTCCTCGAGTTTATAGGTTTCAAGCCGGAATC		
**P14_2F**	5′ – CTCGAGGACTGGGCTGGGAGAATGTCCTGA	Downstream *yusX* gene homologous arm	1023
**P14_2R**	5′ – AAGGCCTTATTGGCCACGCACAGCCAGCTTGATG		
**TS15F**	5′ – TACGTATTAAATCGGCACAGCCGG	**Target sequence for *hrcA* gene deletion**	
**TS15R**	5′ – AAACCCGGCTGTGCCGAATTTAATA		
		**PCR of homology template for *hrcA* gene deletion**	**2019**
**P15_1F**	5′ – AAGGCCAACGAGGCCCGTGCTAAAGCCCTCAAGC	Upstream *hrcA* gene homologous arm	1005
**P15_1R**	5′ – GTCCGAAGTCACCAGCAGCTGACGATTTGTTAAC		
**P15_2F**	5′ – GCTGCTGGTGACTTCGGACTTGTCAAAAG	Downstream *hrcA* gene homologous arm	1014
**P15_2R**	5′ – AAGGCCTTATTGGCCCTTTCAGCGTATGATTTAAGGTG		
**TS16F**	5′ – TACGACGGGATCATTTAAAGATCG	**Target sequence of *thrC* gene for *sipT* gene integration**	
**TS16R**	5′ – AAACCGATCTTTAAATGATCCCGT		
		**PCR of homology template for *thrC* gene replacement for *spiT* gene**	**3369**
**P16_1F**	5′ – AAGGCCAACGAGGCCGATGGGAATTGTGAACGGCAC	Upstream *thrC* gene homologous arm	1017
**P16_1R**	5′ – GCGGACAGTCTGGACAGCATTTAAATGATCCCGTAGGATTGACG		
**P16_2F**	5′ – CGTCAATCCTACGGGATCATTTAAATGCTGTCCAGACTGTCCGC	$P_{amyQ\text{-}cry3a}$ amplification	728
**P16_2R**	5′ – GTATTCGTATTTTTTTCCTCGGTCATGTTTGTCCTCCCTTTCTTATCAT		
**P16_3F**	5′ – ATGATAAGAAAGGGAGGACAAACATGACCGAGGAAAAAAATACGAATAC	*sipT* gene amplification	582
**P16_3R**	5′ – TGCCACAGCCATAACCATTCCTTGGCTATTATCGGTTCAGTGTTCA		
**P16_4F**	5′ – TGAACACTGAACCGATAATAGCCAAGGAATGGTTATGGCTGTGGCA	Downstream *thrC* gene homologous arm	1042
**P16_4R**	5′ – AAGGCCTTATTGGCCTGAATCAAAGCCGGGGCCTA		
		**Gibson assembly primers for *prsA* gene integration at *pel* site**	**8109**
**AF**	5′ – GCTCCTACTGAAAGATTCAGC	Upstream *pel* gene homologous arm	2702
**AR**	5′ – GCCATGTCACTATTGCTTCAGGTCCAAATCATACTCCGGATC		
**BF**	5′ – GATCCGGAGTATGATTTGGACCTGAAGCAATAGTGACATGGC	*erythromycin* resistance gene amplification from pBS2EXxylRPxylA	1721
**BR**	5′ – GTAAAATAATGTCAATGTTGGCTATTCAGGATGGCCTTCTGCTTAG		
**CF**	5′ – CTAAGCAGAAGGCCATCCTGAATAGCCAACATTGACATTATTTTAC	Synthetic *prsA* gene amplification	1064
**CR**	5′ – GATTTCACATTAGCAGAAGCATCTATAAACGCAGAAAGGCCCAC		
**DF**	5′ – GTGGGCCTTTCTGCGTTTATAGATGCTTCTGCTAATGTGAAATC	Downstream *pel* gene homologous arm	2622
**DR**	5′ – CACTTGATCGTTGACGCTTAC		
**pelF**	5′ – GTCTTAGCTCACGACGATGA	PCR for *prsA* integration verification	
**pelR**	5′ – TCCCCTTTTCCATTCTACTCCT		

Name	Sequence	Purpose	PCR Product
		**PCR of homology template for *spoVG* gene replacement for *rasP gene***	**3009**
**P17_1F**	5' – AAGGCCAACGAGGCCCTTGCGAAAAGAAGCATGAAAAC	Upstream *spoVG* gene homologous arm	864
**P17_1R**	5' – CGCTATAACTGTATTCACGAACATAGTAGTTCACCACCTTTTCCC		
**P17_2F**	5' – GGGAAAAGGTGGTGAACTACTATGTTCGTGAATACAGTTATAGCG	*rasP* gene amplification	1269
**P17_2R**	5' – CAGTCCTTGCTTTTTGGTTATTTTTTACAAAAACAGCCGCTGGATAT		
**P17_3F**	5' – ATATCCAGCGGCTGTTTTTGTAAAAAATAACCAAAAAGCAAGGACTG	Downstream *spoVG* gene homologous arm	876
**P17_3R**	5' – AAGGCCTTATTGGCCATTAACTCCGAGCGTTTCTTGG		
**TS18F**	5' – TACGTAACGCAGACAAGGCGATTG	**Target sequence for *yqeZ* replacement**	
**TS18R**	5' – AAACCAATCGCCTTGTCTGCGTTA		
		**PCR of homology template for *yqeZ* gene replacement for *sppA gene***	**2213**
**P18_1F**	5' – AAGGCCAACGAGGCCGGTGGATTTATGAGTCTTCTTGAGC	Upstream *yqeZ* gene homologous arm	586
**P18_1R**	5' – GGGCGGTGTATCCCTCCTTC		
**P18_2F**	5' – GAAGGAGGGATACACCGCCCATGAATGCAAAAAGATGGATTGC	*sppA* gene amplification	1008
**P18_2R**	5' – CATATAACTTCTCCTCGTTTCTATTCTACTTCGCATAGAGATACATC		
**P18_3F**	5' – AATAGAAACGAGGAGAAGTTATATG	Downstream *yqeZ* gene homologous arm	619
**P18_3R**	5' – AAGGCCTTATTGGCCCATGTCAGGGTTTTCAAGCAC		
**TS19F**	5' – TACGTACATTTGACTGGGATACAC	**Target sequence for *sigX* replacement**	
**TS19R**	5' – AAACGTGTATCCCAGTCAAATGTA		
		**PCR of homology template for *sigX* gene replacement for *artificial secYEG operon***	**3476**
**P19_1F**	5' – AAGGCCAACGAGGCCAGTCATGAGCTGAGAACAC	Upstream *sigX* gene homologous arm	856
**P19_1R**	5' – GTTGGAGATTGTTTTAAACAATTGAAACCCCTCCGTTCAC		
**P19_2F**	5' – GTGAACGGAGGGGTTTCAATTGTTTAAAACAATCTCCAACTTTATGC	*secY* gene amplification	1296
**P19_2R**	5' – GTAAAAGACCTCCACAATTTCTAGTTTTTCATAAATCCACGGTAG		
**P19_3F**	5' – CGTGGATTTATGAAAAACTAGAAATTGTGGAGGTCTTTTACATGC	*secE* gene amplification	200
**P19_3R**	5' – CACCTCCAGACTCACTTATTCAACTATTAAACGAATTAATTGAG		
**P19_4F**	5' – TTAATTCGTTTAATAGTTGAATAAGTGAGTCTGGAGGTGTATGG	*secG* gene amplification	252
**P19_4R**	5' – CTGAGGCGAACGATGGTCTTCTATAGGATATAAGCAAGCGCAATC		
**P19_5F**	5' – GCTTGCTTATATCCTATAGAAGACCATCGTTCGCCTCAG	Downstream *sigX* gene homologous arm	872
**P19_5R**	5' – AAGGCCTTATTGGCCAATCATCAACTTCTGACTCC		
**TS20F**	5' – TACGAAAAGAGGTGAAGGCGGTGA	**Target sequence for *gudB* replacement**	
**TS20R**	5' – AAACTCACCGCCTTCACCTCTTTT		
		**PCR of homology template for *gudB* repair**	**1203**
**P20_1F**	5' – AAGGCCAACGAGGCCGAAGATCTAGGGTCACATACG	Upstream *gudB* gene homologous arm	596
**P20_1R**	5' – CCAAATTGAAAGCGCCTTCACCTCTTTTTCTG		
**P20_2F**	5' – GAGGTGAAGGCGCTTTCAATTTGG	Downstream *gudB* gene homologous arm	607
**P20_2R**	5' – AAGGCCTTATTGGCCGAATATCACAATCCAGCTCCAG		

Table S2. Splicing with overlap extension PCR (SOEing-PCR) program.

OVERLAP EXTENSION PCR	TEMPERATURE	TIME
**STEP 1**		
Initial Denaturation	95°C	5 minutes
15 cycles	95°C<br>X °C *<br>72°C	30 seconds<br>1:30 minutes<br>X minutes **
Final Extension	72°C	5 minutes
**STEP 2**		
Initial Denaturation	95°C	5 minutes
30 cycles	95°C<br>X °C *<br>72°C	30 seconds<br>30 seconds<br>X minutes **
Final Extension	72°C	5 minutes
Hold	4 °C	

** Depending on the melting temperature (Tm) of the primer

** Depending on the length of the fragment to be amplified
